# Associations between health- and skill-related physical fitness indicators and cardiometabolic risk factors among Chinese adults: findings from a community-based cross-sectional study

**DOI:** 10.3389/fnut.2026.1718766

**Published:** 2026-03-12

**Authors:** Qishan Ma, Yiqian Lv, Wenjing Liu, Yalei Ke, Meng Feng, Nan Wu, Zhicheng Du, Yuantao Hao, Canqing Yu, Huicui Meng

**Affiliations:** 1 Shenzhen Center for Disease Control and Prevention, Shenzhen, China; 2 School of Public Health (Shenzhen), Sun Yat-Sen University, Shenzhen, China; 3 Department of Epidemiology and Biostatistics, School of Public Health, Peking University Health Science Center, Peking, China; 4 School of Public Health & Center for Health Information Research & Sun Yat-sen Global Health Institute, Sun Yat-sen University, Guangzhou, China; 5 Peking University Center for Public Health and Epidemic Preparedness & Response, Peking, China; 6 Guangdong Provincial Key Laboratory of Food, Nutrition and Health, Guangzhou, China; 7 Guangdong Province Engineering Laboratory for Nutrition Translation, Guangzhou, China

**Keywords:** cardiometabolic risk factor, cross-sectional study, physical activity epidemiology, physical fitness, public health promotion

## Abstract

**Background:**

To determine the relationship between health- and skill-related physical fitness indicators and cardiometabolic risk factors (CMRFs) in Chinese adults.

**Methods:**

A total of 925 participants (483 males and 442 females) in Longhua district, Shenzhen, China were included in this study. Physical fitness assessments were conducted using an all-in-one machine. CMRFs, including blood pressure, fasting plasma glucose concentration, lipid and lipoprotein profiles and uric acid, were measured with standard methods. Linear regression models were used for analysis.

**Results:**

Among health-related physical fitness indicators, body fat mass, body fat percentage and sit-and-reach score were positively associated with diastolic blood pressure (DBP) and/or systolic blood pressure, while step index score was inversely associated with DBP in the fully adjusted models (all *p* < 0.05). Push-up, curl-up, and grip strength scores were not significantly associated with any CMRFs. Among skill-related physical fitness indicators, one-leg standing time was inversely associated with DBP, while choice reaction time was positively associated with concentrations of fasting plasma glucose, total cholesterol, low-density lipoprotein cholesterol (LDL-C), non-high-density lipoprotein cholesterol, and the ratio of LDL-C to high-density lipoprotein cholesterol in the fully adjusted models (all *p* < 0.05). Vertical jump score was not significantly associated with CMRFs.

**Conclusion:**

In conclusion, our study unveils the interplay between various health- and skill-related physical fitness indicators and CMRFs in healthy adults. These findings underscore that improvements in physical fitness, specifically body composition, cardiovascular fitness, and reaction time, were associated with favorable CMRF profiles.

## 1 Introduction

Cardiometabolic diseases (CMDs), including cardiovascular diseases, type 2 diabetes (T2D), and other metabolic diseases, represent a major global health burden with substantial mortality rates in China and worldwide (1, 2). Regular physical activity has been considered an effective strategy in ameliorating cardiometabolic risk factors (CMRFs) (3), while inadequate physical activity is associated with unfavorable CMRFs and increased cardiometabolic risk (4, 5). In addition to the duration and intensity of physical activity, physical fitness, which refers to an individual’s ability to perform various types of physical activity and maintain or enhance physical health, is linked to improved quality of life and reduced cardiometabolic-specific and all-cause mortality (6).

Physical fitness has been defined through a multidimensional hierarchical model with three aspects including physiological fitness, health-related physical fitness (body composition, cardiovascular fitness, flexibility, muscular endurance, and muscle strength), and skill-related physical fitness (agility, balance, coordination, power, speed, and reaction time) (7). Existing research has extensively documented the associations between physiological fitness and health-related physical fitness and CMRFs (8, 9). However, our understanding of the relationships between skill-related physical fitness and CMRFs remains limited. A prospective cohort study observed an inverse association between speed-agility fitness and waist circumference in middle-aged adults (10), while a cross-sectional study has found that vertical jump (VG) was not significantly associated with the risk of dyslipidemia (8). Moreover, a prognostic study showed that walking pace was the only lifestyle variable capable of enhancing the prediction of cardiovascular mortality risk (11). Notably, no study has explored the relationships between other components of skill-related physical fitness, such as balance, coordination, and reaction time, and CMRFs.

With the growing integration of sports medicine into community health systems, comprehensive measurement and documentation of residents’ physical activity and physical fitness will become critical components of future community health management. The primary aim of the present study was to assess the associations between indicators of health-related and skill-related physical fitness and CMRFs, including glucose homeostasis biomarkers, lipid and lipoprotein profiles, blood pressure, and uric acid (UA), in a community-based cross-sectional study of Chinese adults in Longhua district of Shenzhen. The secondary and exploratory aim was to assess the associations in subgroups by sex and age. We hypothesized that better health-related and skill-related physical fitness was associated with favorable CMRFs.

## 2 Methods

### 2.1 Study population

This cross-sectional study enrolled healthy Chinese participants who resided in Longhua district of Shenzhen for at least 1 year. Recruitment of participants was conducted through telephone calls, with interested individuals contacted the designated personnel for further information. Eligible participants were followed up with physical-related notifications, including guidelines, location, and schedule for filling out the physical activity questionnaires, physical fitness examinations, and blood sample collections.

Exclusion criteria encompassed the following situations where individuals were deemed unsuitable for health-related physical fitness evaluation: (1) individuals who suffered from a serious illness and could not complete the survey; (2) individuals with significant cognitive impairment and mental illness; (3) individuals with communication barriers; and (4) unwilling to adhere to the study protocol. The study was conducted between July and August 2021. A total of 1,429 participants were initially selected in the study. The study was conducted in accordance with the guidelines of the Declaration of Helsinki. All procedures were approved by the Ethics Committee of the School of Public Health at Sun Yat-sen University (No. 2017-030), and written informed consent was obtained from all participants.

In the current analysis, participants were excluded if they had missing values in body mass index (BMI) (*n* = 79) or blood pressure (*n* = 56), or did not have any data in any of the physical fitness indicator records (*n* = 136), or did not fill out the questionnaires (*n* = 233). A total of 925 participants were included in the final analysis, including 494 participants of 20–39 years, 385 participants of 40–59 years, and 46 participants of 60–67 years.

### 2.2 Assessment of physical activity status

Physical activity data on the time and intensity (high, moderate or low) of transportation, occupational, household and leisure-time activities as well as the time of sitting were collected via the International Physical Activity Questionnaire (IPAQ), and were expressed as metabolic equivalent of task-minute/week (MET-min/wk) (12). Physical activity status of participants was classified into “high”, “moderate” or “low” (13). The “high” group included those who had vigorous-intensity activities ≥3 days a week (≥1,500 MET-min/wk), or had mixed activities for 7 days (≥3,000 MET-min/wk). The “moderate” group included those who engaged in varieties of vigorous-intensity activities ≥20 min per day for ≥3 days, or engaged in varieties of moderate-intensity and/or walking activities ≥30 min per day for ≥5 days, or had combinations of physical activities at different intensities for ≥5 days per week (≥600 MET-min/wk). Those who failed to meet these criteria were classified as “low” activity.

### 2.3 Assessment of CMRFs

Venous blood samples were collected from each participant following an overnight fast for 8–12 h. Fasting plasma glucose (FPG) concentration was measured by a COBAS8000C702 automatic biochemical instrument (Roche, Germany) with the corresponding reagent. Serum samples were separated by centrifugation at 3,000 g for 15 min, and were immediately stored at −86 °C for subsequent laboratory analysis. Plasma concentrations of total cholesterol (TC), low-density lipoprotein cholesterol (LDL-C), high-density lipoprotein cholesterol (HDL-C), triglyceride (TG), and serum concentrations of UA were measured on the cobas c 702 module (Roche, Germany). The non-high-density lipoprotein cholesterol (non-HDL-C) concentrations and the TC: HDL-C and LDL-C: HDL-C ratios were calculated as the following Equations (1-3):


non-HDL-C=TC-HDL-C (1)

TC:HDL-C=TC/HDL-C (2)

LDL-C:HDL-C=LDL-C/HDL-C (3)

Systolic blood pressure (SBP) and diastolic blood pressure (DBP) were measured after a 15 min seated rest by using an electronic sphygmomanometer (OMRONHEM-7200, Dalian, China). Measures were repeated twice with an interval of 1 min, and the average of the 2 measurements was used in the analysis.

### 2.4 Assessment of physical fitness

Indicators of health-related and skill-related physical fitness were measured on a physical fitness test station (Taishan-TA107, Taishan Sports Technology, Shenzhen, China) by trained research assistants according to the National Physical Fitness Standards Manual (14, 15). The machine was calibrated before each use to ensure accuracy. Indicators of health-related physical fitness included body composition, step index (SP), sit-and-reach (SAR), push-up (PU), curl-up (CU) and grip strength (GS). Indicators of skill-related physical fitness included one-leg standing time (OLST), VG and choice reaction time (RT).

Body composition, including body fat mass (BF) and body fat percentage (BFP), was assessed using bioelectrical impedance analysis with a dedicated body composition analyzer integrated into the Taishan physical fitness testing system (Taishan-TA107). The device has a reported measurement precision of 0.1 kg for body weight and fat mass. Higher values of BF and BFP indicated a higher probability of obesity.

SP was tested only in participants aged < 60 years and assessed by using steps, second chronograph and metronome. During the assessment, participants stood upright in front of the steps and moved up and down the steps according to the sound prompted by the metronome. After 3 min of continuous repetitions, participants immediately sat in a chair and their pulses were recorded at 3 time points, including 1 to 1 and a half minutes, 2 to 2 and a half minutes, and 3 to 3 and a half minutes after the movement stopped. The SP was calculated as the following Equation (4):

Step index=Duration of motion/Sumof three pulses (4)

Higher step index indicated better function of the cardiovascular system.

Flexibility was tested in all participants and evaluated by the SAR test. Participants sat on a pad with legs straight, heels together, toes apart naturally and whole feet on the tester plate. Then participants reached forward with extended arms as far as possible while keeping palms down, arms together and flat, and the upper body forward flexion. The score of SAR was the most distant point reached with their middle fingertips of both hands, and the result was accurate to 0.1 cm. SAR tests were performed twice for each participant, and the higher value was used. Higher SAR values indicated better flexibility of human body.

Among the participants aged between 20 and 39, PU was tested in males and measured by participants according to standard procedures (15) and the total number of PU performed as many as possible was recorded. CU was tested in females according to standard procedures and the total number of CU performed within 1 min was recorded. Greater total number of PU in males indicated better muscle strength and continuous working ability of the upper limbs, shoulders and back of the human body, while greater total number of CU in females indicated better strength and continuous working ability of human waist and abdominal muscles.

GS was tested in all participants and measured by holding the grip dynamometer vigorously to tighten the upper and lower grips with maximum force. GS was accurate to 0.1 kg. GS was measured twice for each participant, and the higher value was used. Higher GS values indicated better muscle strength of the forearm and hand.

Balance performance was tested in all participants and assessed via OLST with eyes closed, which was the time spent standing using one leg on either side while lifting foot of the other side with eyes closed. The test was performed twice for each participant, and the higher value was used. The result was accurate to 0.01 s. Higher OLST values indicated better balance performance.

VG was tested only in participants aged < 60 years and assessed using an electronic vertical jump meter. During the test, participants stood on the vertical jump meter pedal, jumped vertically upwards twice, and the higher value was used. The result was accurate to 0.1 cm. Higher VG values indicated better explosive power of the human body.

RT was tested in all participants and assessed by recording the time period spent on pressing the signal key at the fastest speed when the signal was on and off. The result was accurate to 0.01 s. RT was measured twice for each participant, and the higher value was used. Shorter RT values indicated better coordination and rapid response ability of the nervous and muscular systems.

### 2.5 Assessment of other covariates

Body height and weight of participants were assessed by an all-in-one machine (Taishan-TA107), with height recorded to 0.1 cm and weight to 0.1 kg. The BMI was computed as the ratio of weight to the square of height (kg/m^2^). Sociodemographic data including age and sex were collected with validated questionnaires by trained interviewers.

### 2.6 Statistical analysis

SAS Version 9.4 (SAS Inst., Cary, NC, United States) was used for all statistical analysis, and GraphPad Prism 9.0 (GraphPad Software, La Jolla, CA) and R version 4.3.1 (R Foundation for Statistical Computing, Vienna, Austria) were used for graphs. All variables were tested for normality using the Shapiro–Wilk normality test. Continuous data were described by means ± standard deviation (SD) while count data were described by counts. Data of FPG and TG were analyzed after log transformation. Linear regression models were used to investigate the associations between physical fitness indicators (health-related physical fitness and skill-related physical fitness) and CMRFs (lipid and lipoprotein profiles, glucose homeostasis biomarkers, blood pressure, and UA). Model 1 was a simple linear regression model. Model 2 adjusted for potential confounders, including age (continuous), sex (male or female), and BMI (continuous). Physical activity level (high, moderate, or low) was further adjusted in model 3. In subsequent analyses, data were divided into subgroups on the basis of participants’ sex (male and female) and age (<60 years and ≥60 years), and the associations were assessed in each subgroup with the same confounders in model 2 and model 3. All statistical analyses were two-sided, and statistical significance was accepted at the *p* < 0.05 level.

## 3 Results

### 3.1 Participant characteristics

Table 1 displays the demographic characteristics and physiological and biochemical indicators of the participants. A total of 925 participants (483 males and 442 females) underwent blood biochemical testing. The participants encompassed an age range from 20 to 67 years. The average BMI was 23.65 ± 4.03 kg/m^2^. A majority of the participants were classified as having a high physical activity status (54.7%). Compared with female participants, the BF, BFP, SAR and OLST were lower in male participants. The SAR and GS were lower in participants ≥60 years than in those <60 years. Large inter-individual variations in health and skill-related physical fitness indicators were observed (Table 1; Supplementary Figure S1).

**Table 1:** Demographic characteristics, cardiometabolic risk factors and physical fitness indicators in Chinese adults (*N* = 925).^a^

Characteristics	All	Female	Male	<60 years	≥60 years
*N*	925	442	483	879	46
Age (years)	39.56 ± 11.76	40.02 ± 12.02	39.14 ± 11.51	38.36 ± 10.80	62.35 ± 1.64
BMI (kg/m^2^)	23.65 ± 4.03	22.67 ± 3.22	24.54 ± 4.47	23.59 ± 4.07	24.74 ± 3.01
Physical activity status^b^, *n* (%)					
Low	68 (7.4)	28 (6.3)	40 (8.3)	67 (7.6)	1 (2.2)
Moderate	351 (37.9)	146 (33.1)	205 (42.4)	340 (38.7)	11 (23.9)
High	506 (54.7)	268 (60.6)	238 (49.3)	472 (53.7)	34 (73.9)
Cardiometabolic risk factors^c^					
FPG (mmol/L)	6.46 ± 2.32	6.54 ± 2.23	6.40 ± 2.42	6.34 ± 2.27	6.92 ± 2.52
TC (mmol/L)	5.03 ± 1.05	5.16 ± 1.03	4.92 ± 1.07	4.87 ± 0.99	5.55 ± 1.09
LDL-C (mmol/L)	3.03 ± 0.90	3.17 ± 0.88	2.93 ± 0.91	3.00 ± 0.89	3.14 ± 0.95
non-HDL-C (mmol/L)	3.73 ± 0.98	3.83 ± 0.97	3.64 ± 0.99	3.62 ± 0.97	4.10 ± 0.94
HDL-C (mmol/L)	1.30 ± 0.32	1.32 ± 0.32	1.28 ± 0.31	1.25 ± 0.30	1.45 ± 0.32
TC: HDL-C	3.98 ± 1.03	3.94 ± 0.88	4.00 ± 1.12	3.98 ± 1.04	3.96 ± 1.01
LDL-C: HDL-C	2.42 ± 0.84	2.45 ± 0.74	2.40 ± 0.91	2.49 ± 0.90	2.18 ± 0.56
TG (mmol/L)	1.80 ± 1.65	1.66 ± 0.86	1.91 ± 2.06	1.85 ± 1.80	1.63 ± 0.97
DBP (mmHg)	70.49 ± 10.35	67.84 ± 9.89	72.92 ± 10.18	70.25 ± 10.35	75.06 ± 9.24
SBP (mmHg)	114.57 ± 14.80	109.95 ± 14.99	118.81 ± 13.29	113.86 ± 14.39	128.17 ± 16.07
UA (μmol/L)	377.98 ± 101.57	341.79 ± 96.94	403.44 ± 97.74	382.23 ± 96.99	365.23 ± 115.63
Physical fitness indicators^d^					
Body fat mass (kg)	14.93 ± 5.87	16.32 ± 5.64	13.66 ± 5.79	14.87 ± 5.88	16.03 ± 5.52
Body fat percentage (%)	23.59 ± 7.63	28.57 ± 6.09	18.99 ± 5.82	23.45 ± 7.57	26.08 ± 8.47
Step index	48.99 ± 16.06	52.70 ± 17.64	45.59 ± 13.62	48.99 ± 16.06	NA
Sit-and-reach (cm)	8.15 ± 8.62	9.69 ± 8.04	6.69 ± 8.91	8.31 ± 8.62	4.88 ± 8.02
Push-up (count)	16.35 ± 11.76	NA	16.35 ± 11.76	16.35 ± 11.76	NA
Curl-up (count)	14.80 ± 8.08	14.80 ± 8.08	NA	14.80 ± 8.08	NA
Grip strength (kg)	33.64 ± 10.69	24.84 ± 5.01	41.70 ± 7.75	34.00 ± 10.60	26.63 ± 10.22
One-leg standing time (s)	15.00 ± 20.23	17.56 ± 23.30	12.59 ± 16.50	15.45 ± 20.44	6.04 ± 12.64
Vertical jump (cm)	26.26 ± 8.92	19.96 ± 5.08	31.64 ± 7.91	26.26 ± 8.92	NA
Choice reaction time (s)	0.49 ± 0.10	0.51 ± 0.10	0.48 ± 0.09	0.48 ± 0.09	0.62 ± 0.14

a Data were presented as mean ± SD or *n* (%).

b Physical activity status only included activities with intensity with METs ≥ 2.

c The sample size of each CMRF: FPG, *n* = 110; TC, *n* = 103; LDL-C, *n* = 101; non-HDL-C, *n* = 101; HDL-C, *n* = 101; TC: HDL-C, *n* = 100; LDL-C: HDL-C, *n* = 100; TG, *n* = 103; DBP, *n* = 925; SBP, *n* = 925; UA, *n* = 92.

d The sample size of each physical fitness indicator: Body fat mass, *n* = 887; Body fat percentage, *n* = 887; Step index, *n* = 794; Sit-and-reach, *n* = 906; Push-up, *n* = 234; Curl-up, *n* = 160; Grip strength, *n* = 922; One-leg standing time, *n* = 904; Vertical jump, *n* = 486; Choice reaction time, *n* = 920.

BMI, body mass index; CMRF, cardiometabolic risk factor; DBP, diastolic blood pressure; FPG, fasting plasma glucose; HDL-C, high-density lipoprotein cholesterol; LDL-C, low-density lipoprotein cholesterol; METs, metabolic equivalent of tasks; non-HDL-C, non-high-density lipoprotein cholesterol; NA, not applicable, indicating the corresponding indicators were not assessed in this subgroup; SBP, systolic blood pressure; SD, standard deviation; TC, total cholesterol; TG, triglyceride; UA, uric acid.

### 3.2 Correlations between physical fitness indicators and CMRFs

**Figure 1:**
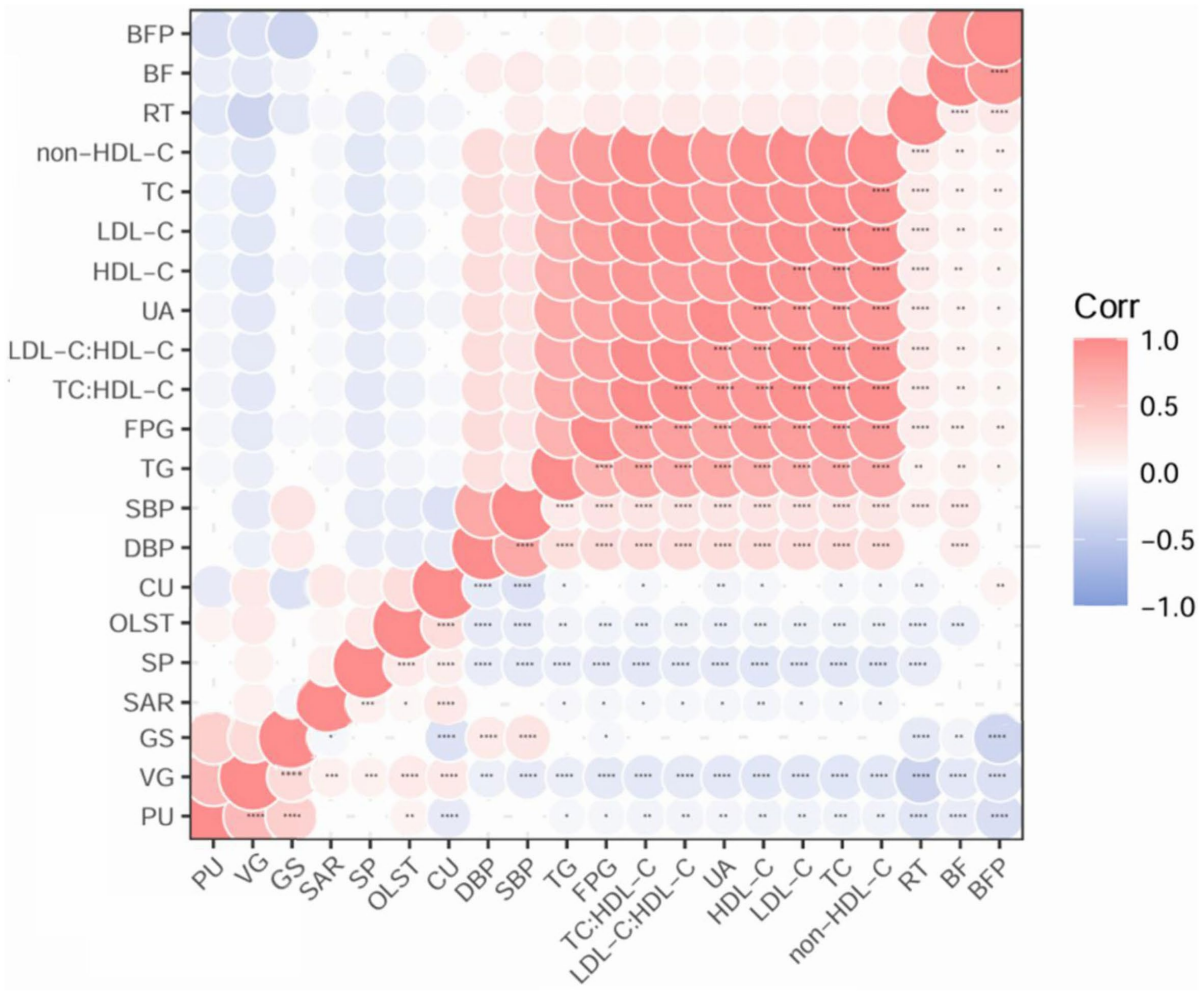
Correlations between physical fitness indicators and cardiometabolic risk factors in a community-based cross-sectional study of Chinese adults. Data was analyzed by Pearson’s or Spearman’s correlation based on normality. Circle size and number indicate the value of correlation coefficient. *p < 0.05; **p < 0.01; ***p < 0.001; ****p < 0.0001. BF, body fat mass; BFP, body fat percentage; Corr, correlation; CU, curl-up; DBP, diastolic blood pressure; FPG, fasting plasma glucose; GS, grip strength; HDL-C, high-density lipoprotein cholesterol; LDL-C, low-density lipoprotein cholesterol; non-HDL-C, non-high-density lipoprotein cholesterol; OLST, one-leg standing time; PU, push-up; RT, choice reaction time; SAR, sit-and-reach; SBP, systolic blood pressure; SP, step index; TC, total cholesterol; TG, triglyceride; UA, uric acid; VG, vertical jump.

BF, BFP, and RT were positively correlated with all CMRFs (*r* = 0.07 to 0.17, all *p* < 0.05). SP, OLST and VG were inversely correlated with all CMRFs (*r* = −0.23 to −0.10, all *p* < 0.05). CU was inversely correlated with DBP (*r* = −0.19, *p* < 0.001) and SBP (*r* = −0.27, *p* < 0.001), while GS was positively correlated with DBP (*r* = 0.18, *p* <0.001) and SBP (*r* = 0.22, *p* < 0.001) (Figure 1). SAR and PU were not significantly correlated with DBP and SBP.

### 3.3 Associations between health-related physical fitness indicators and CMRFs

In fully adjusted models (Model 3), higher BF was associated with higher levels of both DBP [β = 2.18, 95% confidence interval (CI): 0.80, 3.56; *p* = 0.002] and SBP (β = 2.47, 95% CI: 0.61, 4.34; *p* = 0.010), while higher BFP was associated with higher DBP only (β = 2.33, 95% CI: 0.69, 3.97; *p* = 0.005). Higher SP was associated with lower DBP (β = −0.80, 95% CI: −1.49, −0.11; *p* = 0.023). Higher SAR was associated with higher levels of both DBP (β = 0.77, 95% CI: 0.12, 1.41; *p* = 0.020) and SBP (β = 0.97, 95% CI: 0.10, 1.83; *p* = 0.029), respectively. PU, CU and GS were not significantly associated with any CMRFs in all participants (Tables 2–5). Results from crude (Model 1) and partially adjusted models (Model 2) were provided in Supplementary Table S1.

**Table 2:** Associations between physical fitness indicators and blood pressure (*n* = 922).^a^

Physical fitness indicator	DBP (mmHg)		SBP (mmHg)	
	β (95% CI) of Model 3	*p*	β (95% CI) of Model 3	*p*
Health-related fitness				
*Body composition*				
Body fat mass (kg) (*n* = 887)	2.18 (0.80, 3.56)	0.002	2.47 (0.61, 4.34)	0.010
Body fat percentage (%) (*n* = 887)	2.33 (0.69, 3.97)	0.005	1.42 (−0.81, 3.64)	0.21
*Cardiorespiratory fitness*				
Step index (*n* = 794)	−0.80 (−1.49, −0.11)	0.023	−0.72 (−1.61, 0.17)	0.12
*Flexibility*				
Sit-and-reach (cm) (*n* = 906)	0.77 (0.12, 1.41)	0.020	0.97 (0.10, 1.83)	0.029
*Muscular fitness*				
Push-up (count) (*n* = 234)	0.73 (−0.58, 2.04)	0.27	0.35 (−1.20, 1.90)	0.66
Curl-up (count) (*n* = 160)	0.63 (−0.82, 2.08)	0.40	1.62 (−0.23, 3.47)	0.09
Grip strength (kg) (*n* = 922)	0.46 (−0.58, 1.51)	0.38	0.79 (−0.61, 2.19)	0.27
Skill-related fitness				
*Balance*				
One-leg standing time (s) (*n* = 904)	−0.92 (−1.58, −0.27)	0.006	−0.77 (−1.66, 0.11)	0.09
*Power*				
Vertical jump (cm) (*n* = 486)	0.01 (−1.11, 1.14)	0.98	−0.16 (−1.56, 1.23)	0.82
*Reaction time*				
Choice reaction time (s) (*n* = 920)	−0.22 (−0.95, 0.50)	0.54	0.81 (−0.16, 1.78)	0.10

a Model 3 was adjusted for age, sex, body mass index, and physical activity level. The β coefficient represents the estimated change in the blood pressure outcome associated with a one-unit increase in the respective physical fitness indicator.

CI, confidence interval; DBP, diastolic blood pressure; SBP, systolic blood pressure.

**Table 3:** Associations between physical fitness indicators and fasting plasma glucose (*n* = 110).^a^

Physical fitness indicator	FPG (mmol/L)	
	β (95% CI) of Model 3	*p*
Health-related fitness		
*Body composition*		
Body fat mass (kg) (*n* = 106)	−0.02 (−0.18, 0.14)	0.81
Body fat percentage (%) (*n* = 106)	−0.04 (−0.21, 0.13)	0.65
*Cardiorespiratory fitness*		
Step index (*n* = 72)	−0.01 (−0.07, 0.05)	0.77
*Flexibility*		
Sit-and-reach (cm) (*n* = 106)	−0.03 (−0.09, 0.03)	0.30
*Muscular fitness*		
Grip strength (kg) (*n* = 110)	−0.04 (−0.12, 0.05)	0.41
Skill-related fitness		
*Balance*		
One-leg standing time (s) (*n* = 106)	−0.06 (−0.15, 0.02)	0.15
*Reaction time*		
Choice reaction time (s) (*n* = 109)	0.06 (0.01, 0.10)	0.014

a Model 3 was adjusted for age, sex, body mass index, and physical activity level. The β coefficient represents the estimated change in the fasting plasma glucose associated with a one-unit increase in the respective physical fitness indicator. Data for FPG were log-transformed prior to analysis.

CI, confidence interval; FPG, fasting plasma glucose.

**Table 4:** Associations between physical fitness indicators and lipid profiles (*n* = 103).^a^

Physical fitness indicator	β (95% CI) of Model 3	*p*
Health-related fitness		
Body composition		
*Body fat mass (kg)*		
TC (mmol/L) (*n* = 99)	0.40 (−0.23, 1.02)	0.22
LDL-C (mmol/L) (*n* = 97)	0.43 (−0.12, 0.97)	0.13
non-HDL-C (mmol/L) (*n* = 97)	0.36 (−0.24, 0.95)	0.24
HDL-C (mmol/L) (*n* = 97)	0.04 (−0.14, 0.23)	0.65
TC: HDL-C (*n* = 96)	0.27 (−0.35, 0.90)	0.39
LDL-C: HDL-C (*n* = 96)	0.43 (−0.09, 0.94)	0.11
TG (mmol/L) (*n* = 99)	0.16 (−0.07, 0.40)	0.18
*Body fat percentage (%)*		
TC (mmol/L) (*n* = 99)	0.11 (−0.53, 0.75)	0.73
LDL-C (mmol/L) (*n* = 97)	0.16 (−0.41, 0.73)	0.58
non-HDL-C (mmol/L) (*n* = 97)	0.14 (−0.47, 0.75)	0.66
HDL-C (mmol/L) (*n* = 97)	−0.001 (−0.192, 0.190)	0.99
TC: HDL-C (*n* = 96)	0.14 (−0.50, 0.78)	0.67
LDL-C: HDL-C (*n* = 96)	0.24 (−0.29, 0.77)	0.38
TG (mmol/L) (*n* = 99)	0.18 (−0.06, 0.42)	0.14
Cardiorespiratory fitness		
*Step index*		
TC (mmol/L) (*n* = 66)	−0.16 (−0.40, 0.09)	0.22
LDL-C (mmol/L) (*n* = 64)	−0.02 (−0.25, 0.20)	0.85
non-HDL-C (mmol/L) (*n* = 64)	−0.09 (−0.33, 0.14)	0.44
HDL-C (mmol/L) (*n* = 64)	−0.05 (−0.13, 0.03)	0.22
TC: HDL-C (*n* = 63)	−0.01 (−0.26, 0.25)	0.97
LDL-C: HDL-C (*n* = 63)	0.06 (−0.17, 0.29)	0.60
TG (mmol/L) (*n* = 66)	−0.02 (−0.12, 0.09)	0.74
Flexibility		
*Sit-and-reach (cm)*		
TC (mmol/L) (*n* = 99)	0.13 (−0.09, 0.35)	0.24
LDL-C (mmol/L) (*n* = 97)	0.15 (−0.04, 0.35)	0.13
non-HDL-C (mmol/L) (*n* = 97)	0.12 (−0.09, 0.32)	0.28
HDL-C (mmol/L) (*n* = 97)	0.02 (−0.04, 0.09)	0.50
TC: HDL-C (*n* = 96)	0.08 (−0.15, 0.30)	0.52
LDL-C: HDL-C (*n* = 96)	0.07 (−0.12, 0.26)	0.45
TG (mmol/L) (*n* = 99)	−0.002 (−0.087, 0.082)	0.96
Muscular fitness		
*Grip strength (kg)*		
TC (mmol/L) (*n* = 103)	0.07 (−0.25, 0.40)	0.65
LDL-C (mmol/L) (*n* = 101)	0.05 (−0.24, 0.33)	0.75
non-HDL-C (mmol/L) (*n* = 101)	0.12 (−0.18, 0.42)	0.43
HDL-C (mmol/L) (*n* = 101)	−0.05 (−0.15, 0.04)	0.29
TC: HDL-C (*n* = 100)	0.15 (−0.17, 0.47)	0.37
LDL-C: HDL-C (*n* = 100)	0.09 (−0.17, 0.36)	0.49
TG (mmol/L) (*n* = 103)	0.06 (−0.06, 0.18)	0.33
Skill-related fitness		
Balance		
*One-leg standing time (s)*		
TC (mmol/L) (*n* = 98)	0.35 (−0.01, 0.71)	0.06
LDL-C (mmol/L) (*n* = 96)	0.20 (−0.12, 0.53)	0.22
non-HDL-C (mmol/L) (*n* = 96)	0.27 (−0.07, 0.62)	0.13
HDL-C (mmol/L) (*n* = 96)	0.07 (−0.04, 0.18)	0.25
TC: HDL-C (*n* = 95)	0.03 (−0.34, 0.40)	0.88
LDL-C: HDL-C (*n* = 95)	−0.01 (−0.31, 0.30)	0.96
TG (mmol/L) (*n* = 98)	−0.04 (−0.18, 0.10)	0.57
Reaction time		
*Choice reaction time (s)*		
TC (mmol/L) (*n* = 102)	0.21 (0.03, 0.39)	0.022
LDL-C (mmol/L) (*n* = 100)	0.24 (0.08, 0.39)	0.003
non-HDL-C (mmol/L) (*n* = 100)	0.19 (0.02, 0.36)	0.031
HDL-C (mmol/L) (*n* = 100)	0.02 (−0.03, 0.08)	0.42
TC: HDL-C (*n* = 99)	0.10 (−0.09, 0.28)	0.31
LDL-C: HDL-C (*n* = 99)	0.15 (0.00, 0.30)	0.050
TG (mmol/L) (*n* = 102)	−0.03 (−0.10, 0.04)	0.40

a Model 3 was adjusted for age, sex, body mass index, and physical activity level. The β coefficient represents the estimated change in the lipid profiles associated with a one-unit increase in the respective physical fitness indicator. Data for TG were log-transformed prior to analysis.

CI, confidence interval; HDL-C, high-density lipoprotein cholesterol; LDL-C, low-density lipoprotein cholesterol; non-HDL-C, non-high-density lipoprotein cholesterol; TC, total cholesterol; TG, triglyceride.

**Table 5:** Associations between physical fitness indicators and uric acid (*n* = 92).^a^

Physical fitness indicator	Uric acid (μmol/L)	
	β (95% CI) of Model 3	*p*
Health-related fitness		
*Body composition*		
Body fat mass (kg) (*n* = 88)	−5.03 (−67.05, 56.99)	0.87
Body fat percentage (%) (*n* = 88)	−3.55 (−68.96, 61.86)	0.92
*Cardiorespiratory fitness*		
Step index (*n* = 57)	−15.81 (−39.05, 7.44)	0.19
*Flexibility*		
Sit-and-reach (cm) (*n* = 88)	17.63 (−4.32, 39.59)	0.12
*Muscular fitness*		
Grip strength (kg) (*n* = 92)	−2.51 (−34.35, 29.33)	0.88
Skill-related fitness		
*Balance*		
One-leg standing time (s) (*n* = 87)	−14.37 (−49.52, 20.78)	0.43
*Reaction time*		
Choice reaction time (s) (*n* = 91)	−4.46 (−22.28, 13.36)	0.63

a Model 3 was adjusted for age, sex, body mass index, and physical activity level. The β coefficient represents the estimated change in uric acid associated with a one-unit increase in the respective physical fitness indicator.

CI, confidence interval.

### 3.4 Associations between skill-related physical fitness indicators and CMRFs

In the fully adjusted models, higher OLST was associated with lower DBP (β = −0.92, 95% CI: −1.58, −0.27; *p* = 0.006). Higher RT was associated with higher concentrations of FPG and fasting plasma TC, LDL-C, non-HDL-C, and the ratio of LDL-C to HDL-C (FPG: β = 0.06, 95% CI: 0.01, 0.10; *p* = 0.014; TC: β = 0.21, 95% CI: 0.03, 0.39; *p* = 0.022; LDL-C: β = 0.24, 95% CI: 0.08, 0.39; *p* = 0.003; non-HDL-C: β = 0.19, 95% CI: 0.02, 0.36; *p* = 0.031; LDL-C: HDL-C: β = 0.15, 95% CI: 0.00, 0.30; *p* = 0.050), respectively. VG was not significantly associated with any CMRFs in all participants (Tables 2–5). Results from crude (Model 1) and partially adjusted models (Model 2) were provided in Supplementary Table S2.

### 3.5 Associations between physical fitness indicators and CMRFs in subgroups by sex and age

In the subgroup analysis by sex, higher BF was associated with higher levels of both DBP and SBP (DBP: *p* =< 0.001; SBP: *p* = 0.007), and higher BFP was associated with higher DBP only in male participants (*p* = 0.004) (Supplementary Table S3). Conversely, higher SAR was associated with higher fasting plasma LDL-C concentrations (*p* = 0.030) and SBP (*p* = 0.012) only in female participants (Supplementary Table S3), respectively. Higher OLST was associated with lower FPG concentrations (*p* = 0.015) only in female participants, and associated with lower level of DBP (*p* = 0.013) only in male participants (Supplementary Table S4). Higher RT was associated with higher FPG concentrations (*p* = 0.012), TC (*p* = 0.037), LDL-C (*p* = 0.004) and non-HDL-C (*p* = 0.042), and the ratio of LDL-C to HDL-C (*p* = 0.033) only in female participants, and was associated with higher level of SBP (*p* = 0.016) only in male participants (Supplementary Table S4).

In the subgroup analysis by age, higher BF and BFP were associated with higher levels of both DBP and SBP (BF-DBP: *p* = 0.002; BF-SBP: *p* = 0.007; BFP-DBP: *p* = 0.001; BFP-SBP: *p* = 0.019) only in participants aged < 60 years. Higher SAR was associated with higher fasting serum concentrations of UA (*p* = 0.002), while higher GS was associated with lower FPG concentrations (*p* = 0.043) only in participants aged ≥ 60 years (Supplementary Table S5). Higher RT was associated with higher fasting plasma concentrations of LDL-C (*p* = 0.012) only in participants aged < 60 years, and was associated with higher FPG concentrations (*p* = 0.004) only in participants aged ≥ 60 years. Higher OLST was associated with lower levels of DBP (*p* = 0.001) and SBP (*p* = 0.003) only in participants aged < 60 years, and associated with lower DBP (*p* = 0.038) and higher fasting serum concentrations of UA (*p* = 0.006) only in participants aged ≥ 60 years (Supplementary Table S6).

## 4 Discussion

This study comprehensively assessed the relationships between health- and skill-related physical fitness indicators and CMRFs. Results demonstrated large inter-individual variations in the physical fitness indicators among the participants. Health-related physical fitness indicators were associated with blood pressure. Among skill-related physical fitness indicators, balance was associated with DBP, while RT was associated with FPG and lipid profiles. To our knowledge, our study is the first to document the associations between balance and blood pressure, as well as between RT and glucose and lipid profiles.

Body composition indicators (BF and BFP) were positively associated with DBP and/or SBP, consistent with previous research (16). Obesity-related metabolic dysregulation involves sympathetic nervous system activation, endothelial dysfunction and chronic low-grade inflammation responses (17, 18), which collectively contribute to elevated blood pressure and hypertension. Additionally, the prevalence of hypertension is higher in obese adults than in those with normal weight, with independent and positive associations between central obesity and microvascular disease and arterial stiffness risks (18). Therefore, optimizing BF, BFP, and other obesity-related body composition indicators is associated with favorable blood pressure management and hypertension prevention.

Higher SP, indicating better cardiorespiratory fitness, was inversely associated with DBP, in line with a previous study (19). Mechanistically, this relationship may be mediated through enhanced antioxidant enzyme activity, as evidenced by experimental studies showing that elevated cardiorespiratory fitness reduces systemic oxidative stress (20). This physiological adaptation likely contributes to improved endothelial function and blood pressure regulation (21). Evidence indicated that higher levels of cardiorespiratory fitness are associated with the lower risk of developing key CMRFs (22, 23).

Flexibility, measured by SAR test, was positively associated with both SBP and DBP in our participants. SAR was positively associated with plasma concentrations of LDL-C in female participants. Contrary to our results, previous studies have found inverse associations between SAR and the risk of dyslipidemia (8), yet possible explanations for the inconsistent results were not obvious. The generalizability of our findings may be limited by the relatively small sample size, which reduces statistical power to detect smaller effect sizes (*n* = 42) and further research is needed to verify the relationships between flexibility with CMRFs and underlying mechanisms. Future studies with larger and more diverse populations are required to clarify the relationship between flexibility and CMRFs, as well as to elucidate the underlying biological mechanisms.

As an indicator of muscular endurance and muscle strength, GS was inversely associated with FPG concentrations in older adults (≥60 years), suggesting a potential protective role of muscle fitness in glucose regulation. This finding was consistent with analyses based on the China Health and Retirement Longitudinal Study (24). A previous study has demonstrated inverse associations between relative abundances of diabetes-associated gut microbial species and physical fitness, including GS, suggesting that gut microbiota may partially mediate the protective role of physical fitness against T2D (25). Emerging evidence highlights muscle fitness as both an early predictor of CMDs and a therapeutic target for CMD prevention (26). Older adults are more susceptible to a decline in muscle endurance, muscle strength, and sarcopenia (27), which are associated with reduced total energy expenditure, accumulation of overall and ectopic fat, and elevations in pro-inflammatory cytokines (28). These observations underscore that maintaining muscle fitness via physical activity is associated with favorable metabolic health in aging populations.

As an indicator of balance ability, OLST was inversely associated with DBP, specifically in male participants, while inversely associated with FPG concentrations only in female participants. These previously unreported findings suggest potential gender-dimorphic mechanisms warrant further investigation.

RT, as an indirect indicator of neuromuscular coordination and processing speed, was positively associated with fasting plasma concentrations of glucose, TC, LDL-C, non-HDL-C and LDL-C: HDL-C in this study. These observations suggest a link between cognitive function and health-maintenance behaviors. Reaction time, the time between a sensory stimulus and the required response, is significantly associated with cognitive ability (29), with poor cognitive ability are less likely to engage in proper lifestyle and health behaviors (30). Notably, sex-stratified analysis revealed these associations were present exclusively in female participants, representing a novel finding that warrants further investigation into potential sex-specific physiological mechanisms. However, this specific finding, along with results from other sex- and age-stratified analyses, should be interpreted with caution due to the relatively small sample sizes in some subgroups, which may limit statistical power and generalizability. The cross-sectional design precludes causal inference, and the observed associations may be influenced by unmeasured behavioral, cognitive, or lifestyle-related confounding factors.

While our cross-sectional findings did not represent causal relationships, they help identify several potentially modifiable fitness components that may serve as valuable targets for health interventions. Research has demonstrated that structured exercise, such as interval walking training, can effectively enhance muscular strength (31). Similarly, evidence from biomechanical studies has reported that sport-specific training offers a practical pathway to develop agility, balance, and coordination in both recreational and professional settings (32). These evidence-based approaches illustrate that targeted physical activity regimens can be designed to improve the distinct fitness domains associated with metabolic health, providing a practical framework for future intervention strategies.

Our study has several limitations that should be considered when interpreting the findings. First, the cross-sectional design precludes the establishment of temporal sequence and causal inference between physical fitness indicators and CMRFs. Second, the sample sizes for analyses involving biochemical outcomes (glucose, lipids, and uric acid) were substantially smaller than those for blood pressure measurements, which may have reduced statistical power to detect significant associations for these specific outcomes. Third, our study population consisted of a specific cohort of Chinese adults; therefore, the generalizability of our findings to other ethnic groups or populations with different demographic and lifestyle characteristics may be limited. Fourth, while we adjusted for key potential confounders, including age, sex, BMI, and physical activity, the possibility of residual confounding from unmeasured or imprecisely measured behavioral, socioeconomic, or genetic factors cannot be ruled out. Finally, we were unable to investigate the relationships between physical fitness and clinically diagnosed cardiometabolic diseases due to a lack of diagnostic data, and the potential biological mechanisms underlying the observed associations remain unexplored.

## 5 Conclusion

In conclusion, our study identified distinct associations between a range of health- and skill-related physical fitness indicators and cardiometabolic risk factors in a sample of healthy adults. More favorable body composition and cardiovascular fitness were linked to lower blood pressure, while greater flexibility was associated with higher blood pressure. Better balance ability was correlated with lower DBP, and shorter reaction time was related to more favorable glucose and lipid profiles. Some of these associations differed by sex and age. These observations suggest that improving physical fitness attributes, such as body composition, cardiorespiratory capacity, and processing speed, may be associated with improved cardiometabolic health. Future research is needed to confirm these findings in populations at elevated risk for cardiometabolic diseases and to investigate the underlying mechanisms. Such work could inform future strategies for promoting metabolic health through physical activity and fitness in Chinese adults.

## Data Availability

The datasets generated during and analyzed during the current study are not publicly available due to institutional data sharing policies of Shenzhen Center for Disease Control and Prevention, but are available from the corresponding author or first author upon reasonable request. Requests to access these datasets should be directed to Qishan Ma, maqishan@wjw.sz.gov.cn.
