# Supplementary material for: Associations between health- and skill-related physical fitness indicators and cardiometabolic risk factors among Chinese adults: findings from a community-based cross-sectional study
Source: Front Nutr. 2026 Mar 12;13:1718766. doi: 10.3389/fnut.2026.1718766 (PMC13020686; doi:10.3389/fnut.2026.1718766)
Supplement: Supplementary file 1 [file supplementary_file_1.docx]

**Supplemental Table 1.** Associations between health-related physical fitness indicators and cardiometabolic risk factors in Chinese adults (N = 922). ^a^

| **Parameters** | **Model 1** | | | **Model 2** | | |
| --- | --- | --- | --- | --- | --- | --- |
|  | ***β* (95% CI)** | ***P*** | **R^2^ (%)** | ***β* (95% CI)** | ***P*** | **R^2^ (%)** |
| **Body composition** |  |  |  |  |  |  |
| *Body fat mass(kg)* |  |  |  |  |  |  |
| FPG (mmol/L) (n=106) | 0.01 (-0.05, 0.06) | 0.83 | 0.05 | 0.005 (-0.150, 0.160) | 0.95 | 0.91 |
| TC (mmol/L) (n=99) | -0.06 (-0.28, 0.16) | 0.58 | 0.32 | 0.42 (-0.17, 1.01) | 0.17 | 7.60 |
| LDL-C (mmol/L) (n=97) | 0.01 (-0.17, 0.20) | 0.90 | 0.02 | 0.45 (-0.06, 0.96) | 0.09 | 5.31 |
| non-HDL-C (mmol/L) (n=97) | -0.05 (-0.25, 0.16) | 0.66 | 0.20 | 0.31 (-0.25, 0.86) | 0.28 | 4.38 |
| HDL-C (mmol/L) (n=97) | -0.02 (-0.09, 0.05) | 0.55 | 0.38 | 0.10 (-0.08, 0.28) | 0.27 | 7.46 |
| TC: HDL-C (n=96) | -0.02 (-0.23, 0.19) | 0.83 | 0.05 | 0.10 (-0.49, 0.69) | 0.74 | 0.64 |
| LDL-C: HDL-C (n=96) | 0.06 (-0.12, 0.23) | 0.52 | 0.45 | 0.33 (-0.15, 0.81) | 0.18 | 4.42 |
| TG (mmol/L) (n=99) | 0.07 (-0.01, 0.15) | 0.09 | 2.97 | 0.12 (-0.10, 0.34) | 0.30 | 3.56 |
| DBP (mmHg) (n=887) | 1.40 (0.72, 2.07) | <0.001 | 1.83 | 2.25 (0.87, 3.62) | 0.001 | 13.56 |
| SBP (mmHg) (n=887) | 2.62 (1.66, 3.58) | <0.001 | 3.11 | 2.45 (0.58, 4.31) | 0.010 | 23.90 |
| UA (μmol/L) (n=88) | -6.64 (-28.85, 15.58) | 0.56 | 0.40 | -11.43 (-69.49, 46.63) | 0.70 | 9.62 |
| *Body fat percentage (%)* |  |  |  |  |  |  |
| FPG (mmol/L) (n=106) | 0.004 (-0.052, 0.060) | 0.89 | 0.02 | -0.03 (-0.20, 0.13) | 0.69 | 1.07 |
| TC (mmol/L) (n=99) | 0.01 (-0.21, 0.23) | 0.94 | 0.01 | 0.12 (-0.51, 0.74) | 0.72 | 5.83 |
| LDL-C (mmol/L) (n=97) | 0.05 (-0.14, 0.24) | 0.62 | 0.26 | 0.19 (-0.36, 0.73) | 0.51 | 2.75 |
| non-HDL-C (mmol/L) (n=97) | 0.01 (-0.19, 0.22) | 0.92 | 0.01 | 0.09 (-0.50, 0.68) | 0.78 | 3.26 |
| HDL-C (mmol/L) (n=97) | -0.002 (-0.069, 0.064) | 0.94 | 0.01 | 0.05 (-0.14, 0.23) | 0.63 | 6.47 |
| TC: HDL-C (n=96) | -0.06 (-0.27, 0.16) | 0.61 | 0.28 | 0.01 (-0.62, 0.63) | 0.98 | 0.52 |
| LDL-C: HDL-C (n=96) | 0.03 (-0.15, 0.21) | 0.76 | 0.10 | 0.19 (-0.32, 0.70) | 0.47 | 3.09 |
| TG (mmol/L) (n=99) | 0.05 (-0.03, 0.13) | 0.22 | 1.57 | 0.14 (-0.10, 0.37) | 0.25 | 3.80 |
| DBP (mmHg) (n=887) | 0.001 (-0.678, 0.680) | 1.00 | 0 | 2.41 (0.77, 4.05) | 0.004 | 13.38 |
| SBP (mmHg) (n=887) | 0.13 (-0.85, 1.11) | 0.79 | 0.01 | 1.40 (-0.82, 3.62) | 0.22 | 23.46 |
| UA (μmol/L) (n=88) | -20.11 (-42.31, 2.08) | 0.08 | 3.54 | -2.16 (-64.16, 59.84) | 0.95 | 9.46 |
| **Cardiorespiratory fitness** |  |  |  |  |  |  |
| *Step index* |  |  |  |  |  |  |
| FPG (mmol/L) (n=72) | -0.02 (-0.07, 0.04) | 0.61 | 0.37 | -0.01 (-0.07, 0.05) | 0.73 | 4.99 |
| TC (mmol/L) (n=66) | -0.09 (-0.32, 0.15) | 0.48 | 0.78 | -0.17 (-0.42, 0.08) | 0.20 | 6.51 |
| LDL-C (mmol/L) (n=64) | 0.02 (-0.19, 0.23) | 0.83 | 0.07 | -0.03 (-0.25, 0.20) | 0.81 | 3.54 |
| non-HDL-C (mmol/L) (n=64) | -0.05 (-0.28, 0.18) | 0.69 | 0.25 | -0.10 (-0.35, 0.14) | 0.41 | 3.90 |
| HDL-C (mmol/L) (n=64) | -0.03 (-0.10, 0.04) | 0.40 | 1.14 | -0.05 (-0.12, 0.03) | 0.23 | 3.61 |
| TC: HDL-C (n=63) | -0.01 (-0.25, 0.23) | 0.93 | 0.01 | -0.01 (-0.27, 0.24) | 0.91 | 2.99 |
| LDL-C: HDL-C (n=63) | 0.06 (-0.15, 0.27) | 0.58 | 0.50 | 0.06 (-0.17, 0.28) | 0.63 | 2.71 |
| TG (mmol/L) (n=66) | -0.03 (-0.13, 0.06) | 0.50 | 0.71 | -0.02 (-0.12, 0.08) | 0.71 | 4.06 |
| DBP (mmHg) (n=794) | -1.41 (-2.13, -0.70) | <0.001 | 1.87 | -0.81 (-1.50, -0.12) | 0.021 | 13.61 |
| SBP (mmHg) (n=794) | -1.82 (-2.80, -0.83) | <0.001 | 1.63 | -0.72 (-1.61, 0.17) | 0.11 | 24.15 |
| UA (μmol/L) (n=57) | -17.28 (-39.29, 4.74) | 0.13 | 4.12 | -15.61 (-38.82, 7.59) | 0.19 | 10.71 |
| **Flexibility** |  |  |  |  |  |  |
| *Sit-and-reach (cm)* |  |  |  |  |  |  |
| FPG (mmol/L) (n=106) | -0.02 (-0.07, 0.03) | 0.45 | 0.56 | -0.03 (-0.08, 0.03) | 0.32 | 1.93 |
| TC (mmol/L) (n=99) | 0.12 (-0.09, 0.34) | 0.26 | 1.29 | 0.14 (-0.08, 0.36) | 0.22 | 7.15 |
| LDL-C (mmol/L) (n=97) | 0.16 (-0.02, 0.35) | 0.08 | 3.11 | 0.15 (-0.04, 0.35) | 0.12 | 4.65 |
| non-HDL-C (mmol/L) (n=97) | 0.12 (-0.08, 0.33) | 0.23 | 1.52 | 0.13 (-0.08, 0.34) | 0.24 | 4.78 |
| HDL-C (mmol/L) (n=97) | 0.01 (-0.06, 0.07) | 0.82 | 0.05 | 0.02 (-0.05, 0.08) | 0.61 | 5.33 |
| TC: HDL-C (n=96) | 0.08 (-0.13, 0.30) | 0.44 | 0.62 | 0.10 (-0.13, 0.33) | 0.41 | 0.81 |
| LDL-C: HDL-C (n=96) | 0.10 (-0.07, 0.28) | 0.26 | 1.34 | 0.08 (-0.10, 0.27) | 0.39 | 2.40 |
| TG (mmol/L) (n=99) | 0.003 (-0.077, 0.083) | 0.95 | 0 | 0.003 (-0.081, 0.087) | 0.95 | 2.54 |
| DBP (mmHg) (n=906) | 0.03 (-0.64, 0.70) | 0.94 | 0 | 0.77 (0.13, 1.41) | 0.019 | 13.01 |
| SBP (mmHg) (n=906) | -0.52 (-1.48, 0.45) | 0.30 | 0.12 | 0.94 (0.07, 1.80) | 0.034 | 24.42 |
| UA (μmol/L) (n=88) | 10.72 (-10.96, 32.41) | 0.34 | 1.08 | 17.98 (-3.96, 39.92) | 0.11 | 10.22 |
| **Muscular fitness** |  |  |  |  |  |  |
| *Push-up (count)* ^b^ |  |  |  |  |  |  |
| DBP (mmHg) (n=234) | 0.71 (-0.60, 2.02) | 0.29 | 0.48 | 0.74 (-0.56, 2.05) | 0.27 | 2.44 |
| SBP (mmHg) (n=234) | 0.35 (-1.22, 1.91) | 0.67 | 0.08 | 0.33 (-1.22, 1.88) | 0.68 | 3.56 |
| *Curl-up (count)* ^b^ |  |  |  |  |  |  |
| DBP (mmHg) (n=160) | 0.32 (-1.08, 1.73) | 0.65 | 0.13 | 0.59 (-0.85, 2.02) | 0.42 | 2.10 |
| SBP (mmHg) (n=160) | 0.87 (-0.98, 2.72) | 0.36 | 0.54 | 1.56 (-0.27, 3.40) | 0.10 | 7.66 |
| *Grip strength (kg)* |  |  |  |  |  |  |
| FPG (mmol/L) (n=110) | -0.02 (-0.07, 0.03) | 0.47 | 0.48 | -0.04 (-0.12, 0.04) | 0.36 | 1.75 |
| TC (mmol/L) (n=103) | -0.11 (-0.32, 0.10) | 0.30 | 1.07 | 0.07 (-0.25, 0.39) | 0.66 | 6.36 |
| LDL-C (mmol/L) (n=101) | -0.08 (-0.26, 0.10) | 0.39 | 0.76 | 0.04 (-0.24, 0.32) | 0.77 | 2.22 |
| non-HDL-C (mmol/L) (n=101) | -0.06 (-0.25, 0.14) | 0.55 | 0.35 | 0.12 (-0.18, 0.43) | 0.43 | 4.12 |
| HDL-C (mmol/L) (n=101) | -0.05 (-0.11, 0.01) | 0.11 | 2.54 | -0.06 (-0.15, 0.04) | 0.26 | 6.76 |
| TC: HDL-C (n=100) | 0.09 (-0.11, 0.29) | 0.40 | 0.74 | 0.16 (-0.17, 0.48) | 0.35 | 1.06 |
| LDL-C: HDL-C (n=100) | 0.04 (-0.12, 0.21) | 0.61 | 0.27 | 0.10 (-0.17, 0.36) | 0.48 | 2.05 |
| TG (mmol/L) (n=103) | 0.03 (-0.04, 0.11) | 0.43 | 0.62 | 0.06 (-0.06, 0.18) | 0.31 | 3.27 |
| DBP (mmHg) (n=922) | 1.95 (1.30, 2.61) | <0.001 | 3.59 | 0.37 (-0.67, 1.42) | 0.49 | 12.81 |
| SBP (mmHg) (n=922) | 3.38 (2.45, 4.31) | <0.001 | 5.21 | 0.80 (-0.59, 2.20) | 0.26 | 24.42 |
| UA (μmol/L) (n=92) | 20.55 (0.41, 40.70) | 0.049 | 4.25 | -2.79 (-34.65, 29.06) | 0.86 | 9.14 |

^a^ The *β* coefficient represents the estimated change in the CMRF outcome associated with a one-unit increase in the respective health-related physical fitness indicator. Model 1 was an univariable linear regression model. Model 2 was a multivariable linear regression model and adjusted for potential confounders, including age, sex and BMI. Data of FPG and TG were analyzed after log transformation.

^b^ The number of participants in groups of push-up, curl-up with other CMRFs was less than 20, and the sample size was too small for analysis.

Abbreviations: BMI, body mass index; CI, confidence interval; CMRF, cardiometabolic risk factor; DBP, diastolic blood pressure; FPG, fasting plasma glucose; HDL-C, high-density lipoprotein cholesterol; LDL-C, low-density lipoprotein cholesterol; non-HDL-C, non-high-density lipoprotein cholesterol; SBP, systolic blood pressure; TC, total cholesterol; TG, triglyceride; UA, uric acid.

**Supplemental Table 2.** Associations between skill-related physical fitness indicators and cardiometabolic risk factors in Chinese adults (N = 920). ^a^

| **Parameters** | **Model 1** | | | **Model 2** | | |
| --- | --- | --- | --- | --- | --- | --- |
|  | ***β* (95% CI)** | ***P*** | **R^2^ (%)** | ***β* (95% CI)** | ***P*** | **R^2^ (%)** |
| **Balance** |  |  |  |  |  |  |
| *One-leg standing time (s)* |  |  |  |  |  |  |
| FPG (mmol/L) (n=106) | -0.02 (-0.09, 0.05) | 0.62 | 0.24 | -0.04 (-0.12, 0.04) | 0.35 | 1.82 |
| TC (mmol/L) (n=98) | 0.22 (-0.11, 0.54) | 0.19 | 1.75 | 0.38 (0.03, 0.73) | 0.038 | 10.14 |
| LDL-C (mmol/L) (n=96) | 0.18 (-0.10, 0.46) | 0.20 | 1.70 | 0.23 (-0.09, 0.54) | 0.16 | 4.15 |
| non-HDL-C (mmol/L) (n=96) | 0.19 (-0.11, 0.50) | 0.22 | 1.60 | 0.29 (-0.05, 0.63) | 0.10 | 6.11 |
| HDL-C (mmol/L) (n=96) | 0.02 (-0.07, 0.12) | 0.62 | 0.26 | 0.07 (-0.03, 0.18) | 0.19 | 7.38 |
| TC: HDL-C (n=95) | 0.03 (-0.29, 0.35) | 0.87 | 0.03 | 0.02 (-0.35, 0.38) | 0.92 | 0.17 |
| LDL-C: HDL-C (n=95) | 0.06 (-0.21, 0.32) | 0.68 | 0.19 | -0.01 (-0.30, 0.29) | 0.96 | 1.71 |
| TG (mmol/L) (n=98) | -0.04 (-0.16, 0.08) | 0.47 | 0.55 | -0.04 (-0.17, 0.10) | 0.61 | 2.79 |
| DBP (mmHg) (n=904) | -1.82 (-2.48, -1.15) | <0.001 | 3.08 | -0.89 (-1.55, -0.23) | 0.008 | 13.35 |
| SBP (mmHg) (n=904) | -2.77 (-3.72, -1.81) | <0.001 | 3.47 | -0.78 (-1.66, 0.10) | 0.08 | 24.46 |
| UA (μmol/L) (n=87) | -15.35 (-46.35, 15.65) | 0.33 | 1.10 | -18.01 (-52.13, 16.11) | 0.30 | 10.67 |
| **Power** |  |  |  |  |  |  |
| *Vertical jump (cm)* ^b^ |  |  |  |  |  |  |
| DBP (mmHg) (n=486) | 1.76 (0.90, 2.62) | <0.001 | 3.21 | 0.06 (-1.06, 1.19) | 0.91 | 9.69 |
| SBP (mmHg) (n=486) | 3.98 (2.84, 5.12) | <0.001 | 8.80 | -0.18 (-1.57, 1.21) | 0.80 | 26.08 |
| **Reaction time** |  |  |  |  |  |  |
| *Choice reaction time (s)* |  |  |  |  |  |  |
| FPG (mmol/L) (n=109) | 0.04 (0.00, 0.08) | 0.05 | 3.54 | 0.06 (0.01, 0.10) | 0.016 | 6.38 |
| TC (mmol/L) (n=102) | 0.23 (0.07, 0.39) | 0.006 | 7.34 | 0.22 (0.04, 0.39) | 0.018 | 11.07 |
| LDL-C (mmol/L) (n=100) | 0.21 (0.07, 0.35) | 0.003 | 8.59 | 0.24 (0.09, 0.39) | 0.003 | 10.98 |
| non-HDL-C (mmol/L) (n=100) | 0.19 (0.04, 0.34) | 0.013 | 6.12 | 0.20 (0.03, 0.37) | 0.023 | 8.43 |
| HDL-C (mmol/L) (n=100) | 0.03 (-0.02, 0.08) | 0.21 | 1.57 | 0.02 (-0.04, 0.07) | 0.50 | 5.52 |
| TC: HDL-C (n=99) | 0.08 (-0.08, 0.24) | 0.35 | 0.91 | 0.11 (-0.07, 0.29) | 0.24 | 1.58 |
| LDL-C: HDL-C (n=99) | 0.10 (-0.03, 0.24) | 0.12 | 2.43 | 0.16 (0.01, 0.30) | 0.040 | 5.85 |
| TG (mmol/L) (n=102) | -0.01 (-0.07, 0.05) | 0.72 | 0.13 | -0.03 (-0.09, 0.04) | 0.47 | 2.79 |
| DBP (mmHg) (n=920) | 0.56 (-0.10, 1.23) | 0.10 | 0.30 | -0.28 (-1.00, 0.44) | 0.45 | 12.75 |
| SBP (mmHg) (n=920) | 2.52 (1.58, 3.46) | <0.001 | 2.90 | 0.80 (-0.17, 1.76) | 0.11 | 24.64 |
| UA (μmol/L) (n=91) | -8.28 (-24.63, 8.07) | 0.32 | 1.10 | -4.92 (-22.72, 12.88) | 0.59 | 9.06 |

^a^ The *β* coefficient represents the estimated change in the CMRF outcome associated with a one-unit increase in the respective skill-related physical fitness indicator. Model 1 was an univariable linear regression model. Model 2 was a multivariable linear regression model and adjusted for potential confounders, including age, sex and BMI. Data of FPG and TG were analyzed after log transformation.

^b^ The number of participants in groups of vertical jump with other CMRFs was less than 20, and the sample size was too small for analysis.

Abbreviations: BMI, body mass index; CI, confidence interval; CMRF, cardiometabolic risk factor; DBP, diastolic blood pressure; FPG, fasting plasma glucose; HDL-C, high-density lipoprotein cholesterol; LDL-C, low-density lipoprotein cholesterol; non-HDL-C, non-high-density lipoprotein cholesterol; SBP, systolic blood pressure; TC, total cholesterol; TG, triglyceride; UA, uric acid.

**Supplemental Table 3.** Associations between health-related physical fitness indicators and CMRFs in subgroups by sex in Chinese adults (N = 481). ^a^

| **Parameters** | **Model 1** | | | **Model 2** | | | **Model 3** | | |
| --- | --- | --- | --- | --- | --- | --- | --- | --- | --- |
|  | ***β* (95% CI)** | ***P*** | **R^2^ (%)** | ***β* (95% CI)** | ***P*** | **R^2^ (%)** | ***β* (95% CI)** | ***P*** | **R^2^ (%)** |
| **Female** |  |  |  |  |  |  |  |  |  |
| **Body composition** |  |  |  |  |  |  |  |  |  |
| *Body fat mass (kg)* |  |  |  |  |  |  |  |  |  |
| FPG (mmol/L) (n=48) | 0.05 (-0.03, 0.14) | 0.24 | 3.00 | 0.11 (-0.14, 0.37) | 0.39 | 8.27 | 0.10 (-0.15, 0.36) | 0.42 | 14.56 |
| TC (mmol/L) (n=43) | 0.04 (-0.30, 0.38) | 0.81 | 0.15 | 0.74 (-0.30, 1.77) | 0.17 | 7.14 | 0.68 (-0.35, 1.72) | 0.20 | 12.48 |
| LDL-C (mmol/L) (n=41) | 0.04 (-0.26, 0.33) | 0.81 | 0.15 | 0.42 (-0.50, 1.33) | 0.38 | 2.46 | 0.40 (-0.53, 1.33) | 0.41 | 5.28 |
| non-HDL-C (mmol/L) (n=41) | 0.02 (-0.30, 0.34) | 0.91 | 0.04 | 0.44 (-0.56, 1.44) | 0.39 | 2.25 | 0.42 (-0.57, 1.40) | 0.41 | 10.71 |
| HDL-C (mmol/L) (n=41) | 0.01 (-0.09, 0.11) | 0.84 | 0.11 | 0.25 (-0.05, 0.56) | 0.11 | 11.80 | 0.23 (-0.06, 0.53) | 0.13 | 21.54 |
| TC: HDL-C (n=40) | -0.03 (-0.31, 0.25) | 0.85 | 0.09 | -0.15 (-1.03, 0.72) | 0.73 | 1.43 | -0.13 (-0.96, 0.70) | 0.76 | 16.91 |
| LDL-C: HDL-C (n=40) | -0.01 (-0.26, 0.23) | 0.93 | 0.02 | -0.06 (-0.82, 0.69) | 0.87 | 5.58 | -0.03 (-0.75, 0.69) | 0.94 | 18.49 |
| TG (mmol/L) (n=43) | 0.06 (-0.04, 0.16) | 0.28 | 2.82 | -0.09 (-0.40, 0.21) | 0.55 | 11.18 | -0.09 (-0.39, 0.21) | 0.57 | 17.69 |
| DBP (mmHg) (n=426) | 2.29 (1.33, 3.24) | <0.001 | 4.93 | -1.84 (-4.93, 1.25) | 0.24 | 9.70 | -1.80 (-4.90, 1.30) | 0.26 | 10.40 |
| SBP (mmHg) (n=426) | 5.09 (3.67, 6.50) | <0.001 | 10.49 | -3.04 (-7.25, 1.17) | 0.16 | 28.22 | -2.98 (-7.22, 1.25) | 0.17 | 28.28 |
| UA (μmol/L) (n=36) | 9.86 (-24.10, 43.82) | 0.57 | 0.94 | -54.34 (-153.89, 45.21) | 0.29 | 8.12 | -50.67 (-151.39, 50.04) | 0.33 | 9.73 |
| *Body fat percentage (%)* |  |  |  |  |  |  |  |  |  |
| FPG (mmol/L) (n=48) | 0.05 (-0.06, 0.16) | 0.37 | 1.77 | 0.07 (-0.25, 0.38) | 0.68 | 7.05 | 0.04 (-0.27, 0.35) | 0.78 | 13.40 |
| TC (mmol/L) (n=43) | 0.04 (-0.37, 0.44) | 0.86 | 0.08 | 0.62 (-0.65, 1.88) | 0.35 | 4.72 | 0.40 (-0.90, 1.71) | 0.55 | 9.43 |
| LDL-C (mmol/L) (n=41) | 0.004 (-0.349, 0.357) | 0.98 | 0 | 0.19 (-0.92, 1.31) | 0.74 | 0.66 | 0.06 (-1.10, 1.23) | 0.91 | 3.41 |
| non-HDL-C (mmol/L) (n=41) | 0.02 (-0.36, 0.41) | 0.91 | 0.04 | 0.50 (-0.71, 1.71) | 0.42 | 1.98 | 0.29 (-0.94, 1.52) | 0.65 | 9.51 |
| HDL-C (mmol/L) (n=41) | 0.005 (-0.118, 0.128) | 0.94 | 0.01 | 0.16 (-0.22, 0.54) | 0.41 | 7.19 | 0.16 (-0.21, 0.54) | 0.40 | 17.86 |
| TC: HDL-C (n=40) | -0.02 (-0.36, 0.31) | 0.90 | 0.05 | -0.01 (-1.07, 1.05) | 0.99 | 1.11 | -0.15 (-1.18, 0.88) | 0.78 | 16.87 |
| LDL-C: HDL-C (n=40) | -0.04 (-0.33, 0.26) | 0.81 | 0.15 | -0.12 (-1.04, 0.79) | 0.79 | 5.70 | -0.20 (-1.09, 0.70) | 0.67 | 18.92 |
| TG (mmol/L) (n=43) | 0.08 (-0.04, 0.20) | 0.18 | 4.34 | -0.03 (-0.40, 0.34) | 0.88 | 10.40 | -0.06 (-0.44, 0.31) | 0.75 | 17.19 |
| DBP (mmHg) (n=426) | 2.98 (1.83, 4.13) | <0.001 | 5.76 | -0.20 (-3.16, 2.76) | 0.89 | 9.41 | -0.17 (-3.15, 2.80) | 0.91 | 10.12 |
| SBP (mmHg) (n=426) | 6.44 (4.74, 8.13) | <0.001 | 11.54 | -2.17 (-6.20, 1.86) | 0.29 | 28.07 | -2.12 (-6.17, 1.94) | 0.31 | 28.14 |
| UA (μmol/L) (n=36) | 17.81 (-23.95, 59.56) | 0.41 | 2.01 | -27.51 (-148.57, 93.54) | 0.66 | 5.42 | -21.38 (-144.15, 101.39) | 0.74 | 7.25 |
| **Cardiorespiratory fitness** | |  |  |  |  |  |  |  |  |
| *Step index* |  |  |  |  |  |  |  |  |  |
| FPG (mmol/L) (n=32) | -0.08 (-0.18, 0.01) | 0.10 | 8.59 | -0.09 (-0.18, 0.00) | 0.06 | 33.18 | -0.09 (-0.18, 0.00) | 0.06 | 33.20 |
| TC (mmol/L) (n=29) | -0.23 (-0.63, 0.16) | 0.25 | 4.79 | -0.16 (-0.58, 0.25) | 0.44 | 10.31 | -0.11 (-0.53, 0.30) | 0.60 | 19.59 |
| LDL-C (mmol/L) (n=27) | -0.11 (-0.48, 0.25) | 0.55 | 1.42 | -0.06 (-0.45, 0.33) | 0.76 | 5.24 | -0.03 (-0.43, 0.36) | 0.87 | 10.45 |
| non-HDL-C (mmol/L) (n=27) | -0.20 (-0.58, 0.19) | 0.32 | 3.94 | -0.14 (-0.55, 0.26) | 0.50 | 7.89 | -0.10 (-0.49, 0.30) | 0.63 | 22.76 |
| HDL-C (mmol/L) (n=27) | -0.01 (-0.15, 0.14) | 0.92 | 0.04 | 0.0001 (-0.1539, 0.1541) | 1.00 | 0.69 | 0.002 (-0.153, 0.158) | 0.98 | 9.69 |
| TC: HDL-C (n=26) | -0.16 (-0.50, 0.18) | 0.36 | 3.50 | -0.12 (-0.47, 0.24) | 0.52 | 10.36 | -0.07 (-0.42, 0.27) | 0.68 | 24.38 |
| LDL-C: HDL-C (n=26) | -0.06 (-0.34, 0.22) | 0.69 | 0.66 | -0.02 (-0.32, 0.28) | 0.89 | 4.95 | 0.004 (-0.290, 0.297) | 0.98 | 18.64 |
| TG (mmol/L) (n=29) | -0.09 (-0.21, 0.03) | 0.15 | 7.39 | -0.08 (-0.20, 0.04) | 0.18 | 26.31 | -0.08 (-0.19, 0.04) | 0.21 | 33.85 |
| DBP (mmHg) (n=380) | -0.83 (-1.72, 0.06) | 0.07 | 0.88 | -0.71 (-1.57, 0.14) | 0.10 | 9.44 | -0.70 (-1.56, 0.15) | 0.11 | 9.80 |
| SBP (mmHg) (n=380) | -1.01 (-2.31, 0.30) | 0.13 | 0.60 | -0.77 (-1.90, 0.37) | 0.19 | 26.19 | -0.76 (-1.90, 0.37) | 0.19 | 26.26 |
| UA (μmol/L) (n=23) | 4.85 (-31.34, 41.03) | 0.80 | 0.33 | 5.70 (-29.71, 41.10) | 0.76 | 20.79 | 6.82 (-27.85, 41.49) | 0.70 | 28.17 |
| **Flexibility** |  |  |  |  |  |  |  |  |  |
| *Sit-and-reach (cm)* |  |  |  |  |  |  |  |  |  |
| FPG (mmol/L) (n=49) | -0.02 (-0.11, 0.08) | 0.70 | 0.32 | -0.03 (-0.13, 0.06) | 0.51 | 7.84 | -0.04 (-0.13, 0.05) | 0.42 | 14.09 |
| TC (mmol/L) (n=44) | 0.17 (-0.23, 0.57) | 0.41 | 1.64 | 0.26 (-0.17, 0.70) | 0.24 | 5.67 | 0.30 (-0.14, 0.73) | 0.19 | 11.59 |
| LDL-C (mmol/L) (n=42) | 0.37 (0.03, 0.70) | 0.037 | 10.38 | 0.42 (0.04, 0.79) | 0.034 | 11.67 | 0.43 (0.06, 0.81) | 0.030 | 15.31 |
| non-HDL-C (mmol/L) (n=42) | 0.14 (-0.24, 0.52) | 0.48 | 1.27 | 0.19 (-0.23, 0.62) | 0.38 | 2.33 | 0.22 (-0.19, 0.64) | 0.30 | 11.65 |
| HDL-C (mmol/L) (n=42) | 0.07 (-0.05, 0.19) | 0.28 | 2.92 | 0.11 (-0.03, 0.24) | 0.12 | 10.07 | 0.11 (-0.02, 0.24) | 0.11 | 16.58 |
| TC: HDL-C (n=41) | 0.09 (-0.27, 0.44) | 0.64 | 0.57 | 0.10 (-0.31, 0.50) | 0.64 | 1.38 | 0.10 (-0.29, 0.49) | 0.61 | 14.02 |
| LDL-C: HDL-C (n=41) | 0.18 (-0.12, 0.48) | 0.24 | 3.51 | 0.16 (-0.18, 0.50) | 0.35 | 7.20 | 0.15 (-0.18, 0.48) | 0.38 | 17.75 |
| TG (mmol/L) (n=44) | -0.03 (-0.15, 0.09) | 0.65 | 0.49 | -0.03 (-0.16, 0.09) | 0.61 | 10.95 | -0.04 (-0.16, 0.09) | 0.57 | 17.30 |
| DBP (mmHg) (n=439) | 0.33 (-0.67, 1.32) | 0.52 | 0.09 | 0.74 (-0.21, 1.69) | 0.13 | 9.97 | 0.74 (-0.22, 1.69) | 0.13 | 10.80 |
| SBP (mmHg) (n=439) | 0.46 (-1.05, 1.97) | 0.55 | 0.08 | 1.65 (0.36, 2.94) | 0.013 | 28.81 | 1.66 (0.37, 2.96) | 0.012 | 28.92 |
| UA (μmol/L) (n=37) | 25.94 (-16.09, 67.97) | 0.23 | 4.01 | 31.19 (-16.83, 79.21) | 0.21 | 8.82 | 30.58 (-18.27,79.42) | 0.23 | 9.09 |
| **Muscular fitness** | |  |  |  |  |  |  |  |  |
| *Grip strength (kg)* |  |  |  |  |  |  |  |  |  |
| FPG (mmol/L) (n=50) | -0.002 (-0.170, 0.166) | 0.98 | 0 | -0.13 (-0.34, 0.07) | 0.21 | 9.84 | -0.11 (-0.31, 0.09) | 0.30 | 14.81 |
| TC (mmol/L) (n=45) | 0.01 (-0.67, 0.69) | 0.97 | 0 | 0.34 (-0.52, 1.20) | 0.44 | 3.81 | 0.40 (-0.47, 1.27) | 0.38 | 9.49 |
| LDL-C (mmol/L) (n=43) | 0.07 (-0.52, 0.66) | 0.81 | 0.14 | -0.01 (-0.77, 0.75) | 0.97 | 0.46 | -0.02 (-0.81, 0.76) | 0.95 | 3.28 |
| non-HDL-C (mmol/L) (n=43) | 0.06 (-0.59, 0.70) | 0.87 | 0.07 | 0.18 (-0.65, 1.02) | 0.67 | 0.95 | 0.19 (-0.65, 1.02) | 0.66 | 9.57 |
| HDL-C (mmol/L) (n=43) | -0.01 (-0.22, 0.21) | 0.96 | 0.01 | 0.12 (-0.15, 0.38) | 0.40 | 5.22 | 0.16 (-0.12, 0.43) | 0.27 | 11.76 |
| TC: HDL-C (n=42) | -0.09 (-0.69, 0.50) | 0.76 | 0.24 | -0.26 (-1.02, 0.49) | 0.50 | 2.16 | -0.33 (-1.06, 0.41) | 0.39 | 14.46 |
| LDL-C: HDL-C (n=42) | 0.04 (-0.46, 0.54) | 0.88 | 0.06 | -0.26 (-0.88, 0.36) | 0.41 | 6.41 | -0.35 (-0.96, 0.26) | 0.26 | 17.94 |
| TG (mmol/L) (n=45) | -0.14 (-0.34, 0.07) | 0.19 | 3.92 | -0.04 (-0.30, 0.21) | 0.74 | 9.82 | -0.07 (-0.32, 0.19) | 0.60 | 16.07 |
| DBP (mmHg) (n=441) | 0.42 (-1.55, 2.40) | 0.67 | 0.04 | 0.41 (-1.53, 2.34) | 0.68 | 10.02 | 0.55 (-1.39, 2.49) | 0.58 | 10.85 |
| SBP (mmHg) (n=441) | 0.91 (-2.08, 3.90) | 0.55 | 0.08 | 1.67 (-0.95, 4.29) | 0.21 | 28.60 | 1.73 (-0.90, 4.37) | 0.20 | 28.72 |
| UA (μmol/L) (n=38) | -19.23 (-88.09, 49.64) | 0.59 | 0.83 | 0.17 (-85.98, 86.31) | 1.00 | 3.70 | -2.70 (-92.20, 86.79) | 0.95 | 3.94 |
| **Male** |  |  |  |  |  |  |  |  |  |
| **Body composition** |  |  |  |  |  |  |  |  |  |
| *Body fat mass (kg)* |  |  |  |  |  |  |  |  |  |
| FPG (mmol/L) (n=58) | -0.03 (-0.11, 0.04) | 0.39 | 1.34 | -0.12 (-0.33, 0.09) | 0.27 | 2.75 | -0.18 (-0.41, 0.06) | 0.14 | 6.38 |
| TC (mmol/L) (n=56) | -0.20 (-0.50, 0.09) | 0.19 | 3.22 | 0.25 (-0.53, 1.04) | 0.53 | 7.35 | 0.20 (-0.73, 1.13) | 0.68 | 7.90 |
| LDL-C (mmol/L) (n=56) | -0.06 (-0.32, 0.20) | 0.66 | 0.37 | 0.45 (-0.22, 1.13) | 0.20 | 5.06 | 0.36 (-0.45, 1.16) | 0.39 | 5.41 |
| non-HDL-C (mmol/L) (n=56) | -0.15 (-0.43, 0.13) | 0.29 | 2.04 | 0.22 (-0.52, 0.95) | 0.57 | 4.56 | 0.24 (-0.64, 1.11) | 0.60 | 5.35 |
| HDL-C (mmol/L) (n=56) | -0.05 (-0.14, 0.04) | 0.25 | 2.46 | 0.04 (-0.20, 0.27) | 0.76 | 6.81 | -0.04 (-0.32, 0.24) | 0.78 | 8.98 |
| TC: HDL-C (n=56) | 0.0005 (-0.3223, 0.3232) | 1.00 | 0 | 0.22 (-0.65, 1.08) | 0.63 | 0.62 | 0.50 (-0.52, 1.52) | 0.34 | 2.79 |
| LDL-C: HDL-C (n=56) | 0.11 (-0.15, 0.37) | 0.41 | 1.28 | 0.55 (-0.13, 1.24) | 0.12 | 6.13 | 0.66 (-0.15, 1.47) | 0.12 | 6.70 |
| TG (mmol/L) (n=56) | 0.09 (-0.03, 0.22) | 0.15 | 3.81 | 0.26 (-0.07, 0.59) | 0.13 | 7.09 | 0.36 (-0.03, 0.75) | 0.07 | 10.20 |
| DBP (mmHg) (n=461) | 1.95 (1.04, 2.86) | <0.001 | 3.69 | 3.23 (1.51, 4.95) | <0.001 | 6.90 | 3.12 (1.40, 4.85) | <0.001 | 7.34 |
| SBP (mmHg) (n=461) | 2.72 (1.52, 3.92) | <0.001 | 4.11 | 3.12 (0.86, 5.39) | 0.007 | 7.68 | 3.15 (0.88, 5.43) | 0.007 | 7.71 |
| UA (μmol/L) (n=52) | -3.95 (-33.27, 25.36) | 0.79 | 0.14 | 0.12 (-77.30, 77.55) | 1.00 | 0.38 | 12.76 (-78.41, 103.92) | 0.79 | 2.64 |
| *Body fat percentage (%)* |  |  |  |  |  |  |  |  |  |
| FPG (mmol/L) (n=58) | -0.08 (-0.19, 0.04) | 0.21 | 2.85 | -0.18 (-0.40, 0.04) | 0.12 | 4.93 | -0.20 (-0.44, 0.04) | 0.10 | 7.24 |
| TC (mmol/L) (n=56) | -0.34 (-0.78, 0.11) | 0.14 | 3.95 | -0.14 (-0.97, 0.70) | 0.75 | 6.82 | -0.21 (-1.15, 0.72) | 0.65 | 7.96 |
| LDL-C (mmol/L) (n=56) | -0.13 (-0.52, 0.25) | 0.50 | 0.84 | 0.12 (-0.61, 0.85) | 0.75 | 2.11 | -0.03 (-0.84, 0.79) | 0.95 | 3.97 |
| non-HDL-C (mmol/L) (n=56) | -0.28 (-0.69, 0.14) | 0.19 | 3.10 | -0.16 (-0.95, 0.62) | 0.68 | 4.26 | -0.17 (-1.05, 0.70) | 0.70 | 5.10 |
| HDL-C (mmol/L) (n=56) | -0.06 (-0.20, 0.08) | 0.39 | 1.40 | 0.03 (-0.23, 0.28) | 0.85 | 6.71 | -0.04 (-0.32, 0.24) | 0.78 | 8.98 |
| TC: HDL-C (n=56) | -0.06 (-0.55, 0.42) | 0.80 | 0.12 | -0.05 (-0.97, 0.87) | 0.92 | 0.19 | 0.09 (-0.94, 1.13) | 0.86 | 1.07 |
| LDL-C: HDL-C (n=56) | 0.11 (-0.28, 0.50) | 0.59 | 0.54 | 0.31 (-0.43, 1.05) | 0.41 | 2.82 | 0.29 (-0.54, 1.12) | 0.50 | 2.84 |
| TG (mmol/L) (n=56) | 0.14 (-0.05, 0.32) | 0.16 | 3.70 | 0.25 (-0.10, 0.60) | 0.17 | 6.44 | 0.35 (-0.04, 0.73) | 0.08 | 9.76 |
| DBP (mmHg) (n=461) | 2.52 (1.35, 3.70) | <0.001 | 3.70 | 3.44 (1.22, 5.66) | 0.003 | 6.03 | 3.33 (1.10, 5.56) | 0.004 | 6.54 |
| SBP (mmHg) (n=461) | 3.21 (1.65, 4.77) | <0.001 | 3.42 | 1.81 (-1.11, 4.74) | 0.23 | 6.51 | 1.82 (-1.13, 4.77) | 0.23 | 6.52 |
| UA (μmol/L) (n=52) | -8.63 (-53.05, 35.80) | 0.71 | 0.29 | -7.30 (-90.86, 76.27) | 0.86 | 0.44 | -10.08 (-102.64, 82.48) | 0.83 | 2.57 |
| **Cardiorespiratory fitness** | |  |  |  |  |  |  |  |  |
| *Step index* |  |  |  |  |  |  |  |  |  |
| FPG (mmol/L) (n=40) | 0.02 (-0.06, 0.09) | 0.69 | 0.41 | 0.01 (-0.08, 0.10) | 0.83 | 1.62 | 0.01 (-0.08, 0.10) | 0.87 | 8.22 |
| TC (mmol/L) (n=37) | -0.04 (-0.36, 0.27) | 0.79 | 0.20 | -0.13 (-0.49, 0.23) | 0.49 | 3.40 | -0.15 (-0.51, 0.22) | 0.44 | 9.88 |
| LDL-C (mmol/L) (n=37) | 0.08 (-0.19, 0.36) | 0.56 | 0.97 | 0.03 (-0.29, 0.35) | 0.86 | 3.49 | 0.01 (-0.31, 0.33) | 0.96 | 8.22 |
| non-HDL-C (mmol/L) (n=37) | 0.02 (-0.29, 0.32) | 0.92 | 0.03 | -0.04 (-0.40, 0.31) | 0.81 | 1.81 | -0.06 (-0.41, 0.29) | 0.75 | 10.29 |
| HDL-C (mmol/L) (n=37) | -0.06 (-0.14, 0.02) | 0.16 | 5.48 | -0.08 (-0.18, 0.01) | 0.08 | 9.23 | -0.09 (-0.18, 0.01) | 0.07 | 13.43 |
| TC: HDL-C (n=37) | 0.12 (-0.22, 0.45) | 0.50 | 1.34 | 0.12 (-0.28, 0.51) | 0.57 | 1.71 | 0.12 (-0.28, 0.52) | 0.56 | 5.95 |
| LDL-C: HDL-C (n=37) | 0.16 (-0.15, 0.46) | 0.32 | 2.82 | 0.16 (-0.20, 0.51) | 0.38 | 3.74 | 0.15 (-0.22, 0.52) | 0.43 | 4.84 |
| TG (mmol/L) (n=37) | 0.02 (-0.12, 0.17) | 0.76 | 0.27 | 0.05 (-0.12, 0.22) | 0.59 | 1.32 | 0.05 (-0.13, 0.22) | 0.60 | 4.76 |
| DBP (mmHg) (n=414) | -0.96 (-2.12, 0.20) | 0.11 | 0.63 | -0.96 (-2.10, 0.18) | 0.10 | 6.78 | -0.93 (-2.07, 0.20) | 0.11 | 7.18 |
| SBP (mmHg) (n=414) | -0.62 (-2.07, 0.83) | 0.40 | 0.17 | -0.52 (-1.93, 0.89) | 0.47 | 7.48 | -0.51 (-1.93, 0.90) | 0.48 | 7.57 |
| UA (μmol/L) (n=34) | -23.17 (-51.48, 5.14) | 0.12 | 7.44 | -33.13 (-65.11, -1.15) | 0.05 | 13.48 | -29.88 (-63.08, 3.32) | 0.09 | 16.70 |
| **Flexibility** |  |  |  |  |  |  |  |  |  |
| *Sit-and-reach (cm)* |  |  |  |  |  |  |  |  |  |
| FPG (mmol/L) (n=57) | -0.03 (-0.10, 0.04) | 0.42 | 1.19 | -0.03 (-0.11, 0.04) | 0.39 | 1.61 | -0.04 (-0.11, 0.04) | 0.33 | 4.26 |
| TC (mmol/L) (n=55) | 0.06 (-0.21, 0.33) | 0.65 | 0.39 | 0.10 (-0.17, 0.38) | 0.47 | 6.31 | 0.08 (-0.20, 0.36) | 0.58 | 9.96 |
| LDL-C (mmol/L) (n=55) | 0.05 (-0.18, 0.28) | 0.67 | 0.35 | 0.07 (-0.17, 0.31) | 0.59 | 1.57 | 0.06 (-0.19, 0.30) | 0.65 | 4.19 |
| non-HDL-C (mmol/L) (n=55) | 0.08 (-0.16, 0.33) | 0.51 | 0.84 | 0.12 (-0.14, 0.38) | 0.38 | 4.44 | 0.09 (-0.17, 0.35) | 0.50 | 9.24 |
| HDL-C (mmol/L) (n=55) | -0.02 (-0.10, 0.06) | 0.59 | 0.57 | -0.01 (-0.10, 0.07) | 0.73 | 5.76 | -0.01 (-0.09, 0.07) | 0.80 | 7.06 |
| TC: HDL-C (n=55) | 0.10 (-0.19, 0.39) | 0.51 | 0.83 | 0.12 (-0.19, 0.42) | 0.45 | 1.31 | 0.10 (-0.21, 0.41) | 0.54 | 3.40 |
| LDL-C: HDL-C (n=55) | 0.07 (-0.17, 0.30) | 0.57 | 0.62 | 0.07 (-0.18, 0.31) | 0.59 | 1.38 | 0.06 (-0.19, 0.32) | 0.62 | 1.72 |
| TG (mmol/L) (n=55) | 0.02 (-0.09, 0.13) | 0.76 | 0.18 | 0.01 (-0.11, 0.13) | 0.85 | 3.12 | 0.004 (-0.116, 0.124) | 0.95 | 4.88 |
| DBP (mmHg) (n=467) | 0.59 (-0.30, 1.48) | 0.19 | 0.36 | 0.80 (-0.08, 1.67) | 0.07 | 5.84 | 0.79 (-0.09, 1.67) | 0.08 | 6.13 |
| SBP (mmHg) (n=467) | 0.09 (-1.09, 1.26) | 0.89 | 0 | 0.41 (-0.73, 1.55) | 0.48 | 8.06 | 0.44 (-0.71, 1.58) | 0.45 | 8.10 |
| UA (μmol/L) (n=51) | 14.19 (-10.81, 39.19) | 0.27 | 2.46 | 12.30 (-14.12, 38.71) | 0.37 | 3.04 | 13.45 (-13.29,40.18) | 0.33 | 6.59 |
| **Muscular fitness** | |  |  |  |  |  |  |  |  |
| *Grip strength (kg)* |  |  |  |  |  |  |  |  |  |
| FPG (mmol/L) (n=60) | -0.02 (-0.11, 0.06) | 0.58 | 0.52 | -0.03 (-0.12, 0.06) | 0.53 | 0.97 | -0.03 (-0.13, 0.06) | 0.53 | 2.75 |
| TC (mmol/L) (n=58) | -0.06 (-0.41, 0.28) | 0.73 | 0.22 | 0.03 (-0.33, 0.38) | 0.88 | 6.90 | 0.02 (-0.33, 0.38) | 0.90 | 8.94 |
| LDL-C (mmol/L) (n=58) | 0.001 (-0.293, 0.295) | 1.00 | 0 | 0.04 (-0.27, 0.35) | 0.80 | 1.75 | 0.04 (-0.28, 0.35) | 0.82 | 4.18 |
| non-HDL-C (mmol/L) (n=58) | 0.04 (-0.28, 0.36) | 0.81 | 0.11 | 0.11 (-0.22, 0.45) | 0.51 | 4.76 | 0.11 (-0.23, 0.44) | 0.53 | 7.26 |
| HDL-C (mmol/L) (n=58) | -0.10 (-0.20, 0.00) | 0.046 | 6.93 | -0.08 (-0.19, 0.02) | 0.11 | 11.13 | -0.09 (-0.19, 0.02) | 0.11 | 13.24 |
| TC: HDL-C (n=58) | 0.20 (-0.16, 0.56) | 0.29 | 1.99 | 0.23 (-0.16, 0.61) | 0.25 | 2.54 | 0.23 (-0.16, 0.61) | 0.26 | 4.30 |
| LDL-C: HDL-C (n=58) | 0.16 (-0.14, 0.45) | 0.30 | 1.95 | 0.15 (-0.16, 0.46) | 0.35 | 2.38 | 0.15 (-0.17, 0.47) | 0.36 | 2.70 |
| TG (mmol/L) (n=58) | 0.10 (-0.04, 0.24) | 0.16 | 3.47 | 0.09 (-0.06, 0.23) | 0.25 | 5.39 | 0.09 (-0.06, 0.23) | 0.26 | 6.74 |
| DBP (mmHg) (n=481) | -0.22 (-1.47, 1.03) | 0.73 | 0.02 | 0.28 (-0.99, 1.54) | 0.67 | 5.45 | 0.33 (-0.94, 1.60) | 0.61 | 5.84 |
| SBP (mmHg) (n=481) | -0.71 (-2.36, 0.93) | 0.39 | 0.15 | -0.004 (-1.643, 1.635) | 1.00 | 8.00 | -0.002 (-1.647, 1.643) | 1.00 | 8.06 |
| UA (μmol/L) (n=54) | -0.38 (-33.31, 32.54) | 0.98 | 0 | -3.25 (-38.16, 31.67) | 0.86 | 0.95 | -1.57 (-36.83, 33.69) | 0.93 | 3.81 |

^a^ The *β* coefficient represents the estimated change in the CMRF outcome associated with a one-unit increase in the respective health-related physical fitness indicator. Model 1 was an univariable linear regression model. Model 2 was a multivariable linear regression model and adjusted for potential confounders, including age, sex and BMI. Model 3 further adjusted for physical activity on the basis of model 2. Data of FPG and TG were analyzed after log transformation.

Abbreviations: BMI, body mass index; CI, confidence interval; CMRF, cardiometabolic risk factors; DBP, diastolic blood pressure; FPG, fasting plasma glucose; HDL-C, high-density lipoprotein cholesterol; LDL-C, low-density lipoprotein cholesterol; non-HDL-C, non-high-density lipoprotein cholesterol; SBP, systolic blood pressure; TC, total cholesterol; TG, triglyceride; UA, uric acid.

**Supplemental Table 4.** Associations between skill-related physical fitness indicators and CMRFs in subgroups by sex in Chinese adults (N = 478). ^a^

| **Parameters** | **Model 1** | | | **Model 2** | | | **Model 3** | | |
| --- | --- | --- | --- | --- | --- | --- | --- | --- | --- |
|  | ***β* (95% CI)** | ***P*** | **R^2^ (%)** | ***β* (95% CI)** | ***P*** | **R^2^ (%)** | ***β* (95% CI)** | ***P*** | **R^2^ (%)** |
| **Female** |  |  |  |  |  |  |  |  |  |
| **Balance** |  |  |  |  |  |  |  |  |  |
| *One-leg standing time (s)* | |  |  |  |  |  |  |  |  |
| FPG (mmol/L) (n=50) | -0.03 (-0.11, 0.04) | 0.39 | 1.51 | -0.06 (-0.14, 0.03) | 0.19 | 10.22 | -0.11 (-0.20, -0.03) | 0.015 | 23.51 |
| TC (mmol/L) (n=45) | 0.19 (-0.15, 0.53) | 0.27 | 2.79 | 0.33 (-0.06, 0.72) | 0.10 | 8.55 | 0.28 (-0.15, 0.71) | 0.21 | 11.37 |
| LDL-C (mmol/L) (n=43) | 0.17 (-0.12, 0.46) | 0.26 | 3.11 | 0.20 (-0.15, 0.55) | 0.27 | 3.58 | 0.19 (-0.19, 0.57) | 0.34 | 5.69 |
| non-HDL-C (mmol/L) (n=43) | 0.15 (-0.17, 0.47) | 0.36 | 2.05 | 0.21 (-0.17, 0.60) | 0.28 | 3.40 | 0.15 (-0.26, 0.56) | 0.47 | 10.42 |
| HDL-C (mmol/L) (n=43) | 0.04 (-0.07, 0.14) | 0.48 | 1.25 | 0.09 (-0.03, 0.21) | 0.15 | 8.43 | 0.10 (-0.04, 0.23) | 0.16 | 13.53 |
| TC: HDL-C (n=42) | -0.03 (-0.32, 0.27) | 0.87 | 0.07 | -0.10 (-0.45, 0.25) | 0.57 | 1.81 | -0.17 (-0.54, 0.19) | 0.36 | 14.74 |
| LDL-C: HDL-C (n=42) | 0.03 (-0.22, 0.28) | 0.81 | 0.14 | -0.07 (-0.36, 0.22) | 0.65 | 5.23 | -0.08 (-0.38, 0.23) | 0.61 | 15.60 |
| TG (mmol/L) (n=45) | -0.07 (-0.17, 0.03) | 0.17 | 4.36 | -0.02 (-0.14, 0.09) | 0.69 | 9.93 | -0.03 (-0.16, 0.10) | 0.65 | 15.92 |
| DBP (mmHg) (n=439) | -1.33 (-2.13, -0.54) | 0.001 | 2.41 | -0.55 (-1.36, 0.26) | 0.18 | 10.02 | -0.58 (-1.39, 0.23) | 0.16 | 10.87 |
| SBP (mmHg) (n=439) | -2.66 (-3.86, -1.47) | <0.001 | 4.17 | -0.54 (-1.63, 0.56) | 0.34 | 27.99 | -0.54 (-1.65, 0.56) | 0.33 | 28.09 |
| UA (μmol/L) (n=38) | -18.35 (-50.19, 13.49) | 0.27 | 3.42 | -11.08 (-51.35, 29.20) | 0.59 | 4.52 | -10.24 (-53.24, 32.77) | 0.64 | 4.56 |
| **Power** |  |  |  |  |  |  |  |  |  |
| *Vertical jump (cm)* ^b^ |  |  |  |  |  |  |  |  |  |
| DBP (mmHg) (n=224) | 0.63 (-1.39, 2.66) | 0.54 | 0.17 | 0.80 (-1.24, 2.85) | 0.44 | 2.27 | 0.81 (-1.24, 2.86) | 0.44 | 3.37 |
| SBP (mmHg) (n=224) | 1.08 (-1.51, 3.68) | 0.41 | 0.30 | 1.60 (-0.98, 4.17) | 0.23 | 5.96 | 1.70 (-0.88, 4.28) | 0.20 | 6.68 |
| **Reaction time** |  |  |  |  |  |  |  |  |  |
| *Choice reaction time (s)* |  |  |  |  |  |  |  |  |  |
| FPG (mmol/L) (n=50) | 0.05 (0.00, 0.10) | 0.07 | 6.66 | 0.07 (0.01, 0.12) | 0.015 | 18.15 | 0.07 (0.02, 0.12) | 0.012 | 24.38 |
| TC (mmol/L) (n=45) | 0.23 (0.04, 0.42) | 0.021 | 11.74 | 0.24 (0.03, 0.45) | 0.029 | 13.28 | 0.23 (0.02, 0.44) | 0.037 | 17.48 |
| LDL-C (mmol/L) (n=43) | 0.22 (0.06, 0.38) | 0.009 | 15.58 | 0.27 (0.10, 0.44) | 0.003 | 20.44 | 0.27 (0.10, 0.44) | 0.004 | 22.61 |
| non-HDL-C (mmol/L) (n=43) | 0.19 (0.01, 0.37) | 0.044 | 9.54 | 0.22 (0.03, 0.45) | 0.036 | 11.26 | 0.21 (0.01, 0.41) | 0.042 | 18.84 |
| HDL-C (mmol/L) (n=43) | 0.03 (-0.03, 0.09) | 0.34 | 2.25 | 0.02 (-0.05, 0.09) | 0.53 | 4.44 | 0.02 (-0.05, 0.09) | 0.55 | 9.69 |
| TC: HDL-C (n=42) | 0.08 (-0.09, 0.25) | 0.36 | 2.09 | 0.12 (-0.07, 0.30) | 0.23 | 4.69 | 0.11 (-0.07, 0.29) | 0.24 | 15.96 |
| LDL-C: HDL-C (n=42) | 0.11 (-0.04, 0.25) | 0.15 | 5.07 | 0.16 (0.01, 0.31) | 0.039 | 14.98 | 0.16 (0.02, 0.31) | 0.033 | 25.17 |
| TG (mmol/L) (n=45) | 0.02 (-0.04, 0.08) | 0.56 | 0.82 | -0.01 (-0.07, 0.06) | 0.83 | 9.66 | -0.01 (-0.07, 0.06) | 0.83 | 15.55 |
| DBP (mmHg) (n=442) | 1.15 (0.26, 2.04) | 0.012 | 1.42 | -0.24 (-1.22, 0.74) | 0.63 | 9.62 | -0.18 (-1.16, 0.80) | 0.72 | 10.41 |
| SBP (mmHg) (n=442) | 3.58 (2.26, 4.90) | <0.001 | 6.01 | -0.09 (-1.41, 1.24) | 0.90 | 27.81 | -0.05 (-1.39, 1.28) | 0.94 | 27.91 |
| UA (μmol/L) (n=38) | -0.11 (-20.02, 19.80) | 0.99 | 0 | -4.46 (-25.70, 16.78) | 0.68 | 4.18 | -4.21 (-25.85, 17.42) | 0.71 | 4.36 |
| **Male** |  |  |  |  |  |  |  |  |  |
| **Balance** |  |  |  |  |  |  |  |  |  |
| *One-leg standing time (s)* | |  |  |  |  |  |  |  |  |
| FPG (mmol/L) (n=56) | 0.07 (-0.14, 0.28) | 0.52 | 0.77 | 0.08 (-0.17, 0.33) | 0.53 | 1.00 | 0.09 (-0.16, 0.34) | 0.50 | 3.45 |
| TC (mmol/L) (n=53) | 0.30 (-0.63, 1.23) | 0.53 | 0.76 | 0.79 (-0.21, 1.79) | 0.13 | 10.86 | 0.82 (-0.19, 1.84) | 0.12 | 12.81 |
| LDL-C (mmol/L) (n=53) | 0.17 (-0.63, 0.97) | 0.67 | 0.35 | 0.38 (-0.52, 1.27) | 0.41 | 2.78 | 0.37 (-0.54, 1.28) | 0.43 | 4.44 |
| non-HDL-C (mmol/L) (n=53) | 0.38 (-0.49, 1.25) | 0.39 | 1.43 | 0.76 (-0.18, 1.71) | 0.12 | 8.33 | 0.82 (-0.14, 1.77) | 0.10 | 11.27 |
| HDL-C (mmol/L) (n=53) | -0.08 (-0.36, 0.19) | 0.56 | 0.68 | 0.02 (-0.28, 0.32) | 0.87 | 6.96 | 0.01 (-0.30, 0.31) | 0.96 | 8.83 |
| TC: HDL-C (n=53) | 0.40 (-0.58, 1.39) | 0.43 | 1.24 | 0.54 (-0.57, 1.66) | 0.35 | 1.88 | 0.61 (-0.52, 1.74) | 0.29 | 4.46 |
| LDL-C: HDL-C (n=53) | 0.20 (-0.59, 1.00) | 0.62 | 0.49 | 0.14 (-0.76, 1.04) | 0.76 | 0.97 | 0.14 (-0.78, 1.07) | 0.76 | 1.24 |
| TG (mmol/L) (n=53) | 0.15 (-0.23, 0.54) | 0.44 | 1.16 | 0.07 (-0.36, 0.50) | 0.75 | 3.86 | 0.09 (-0.35, 0.52) | 0.69 | 5.88 |
| DBP (mmHg) (n=465) | -1.92 (-3.05, -0.79) | <0.001 | 2.35 | -1.41 (-2.54, -0.28) | 0.015 | 6.98 | -1.43 (-2.55, -0.30) | 0.013 | 7.58 |
| SBP (mmHg) (n=465) | -1.51 (-2.99, -0.02) | 0.048 | 0.85 | -0.66 (-2.12, 0.80) | 0.38 | 8.37 | -0.66 (-2.12, 0.81) | 0.38 | 8.40 |
| UA (μmol/L) (n=49) | 23.55 (-59.49, 106.58) | 0.58 | 0.65 | 1.36 (-92.32, 95.04) | 0.98 | 3.27 | 0.45 (-94.68, 95.58) | 0.99 | 5.58 |
| **Power** |  |  |  |  |  |  |  |  |  |
| *Vertical jump (cm)* ^b^ |  |  |  |  |  |  |  |  |  |
| DBP (mmHg) (n=262) | -0.44 (-1.80, 0.91) | 0.52 | 0.16 | -0.17 (-1.55, 1.21) | 0.81 | 1.82 | -0.24 (-1.63, 1.14) | 0.73 | 2.62 |
| SBP (mmHg) (n=262) | -0.95 (-2.61, 0.72) | 0.27 | 0.47 | -0.88 (-2.56, 0.80) | 0.30 | 4.27 | -0.92 (-2.61, 0.77) | 0.29 | 4.54 |
| **Reaction time** |  |  |  |  |  |  |  |  |  |
| *Choice reaction time (s)* |  |  |  |  |  |  |  |  |  |
| FPG (mmol/L) (n=59) | 0.03 (-0.05, 0.11) | 0.45 | 1.00 | 0.05 (-0.04, 0.14) | 0.30 | 2.20 | 0.05 (-0.05, 0.15) | 0.31 | 3.99 |
| TC (mmol/L) (n=57) | 0.19 (-0.10, 0.48) | 0.21 | 2.89 | 0.17 (-0.17, 0.50) | 0.34 | 7.28 | 0.17 (-0.18, 0.52) | 0.36 | 8.80 |
| LDL-C (mmol/L) (n=57) | 0.15 (-0.10, 0.40) | 0.25 | 2.42 | 0.17 (-0.13, 0.46) | 0.27 | 3.46 | 0.19 (-0.11, 0.50) | 0.22 | 6.01 |
| non-HDL-C (mmol/L) (n=57) | 0.16 (-0.11, 0.44) | 0.25 | 2.43 | 0.15 (-0.17, 0.50) | 0.35 | 4.79 | 0.14 (-0.19, 0.47) | 0.40 | 6.76 |
| HDL-C (mmol/L) (n=57) | 0.03 (-0.06, 0.11) | 0.54 | 0.69 | 0.01 (-0.09, 0.11) | 0.80 | 5.93 | 0.02 (-0.08, 0.13) | 0.65 | 7.97 |
| TC: HDL-C (n=57) | 0.09 (-0.22, 0.41) | 0.57 | 0.59 | 0.10 (-0.28, 0.47) | 0.62 | 0.59 | 0.06 (-0.33, 0.45) | 0.75 | 2.06 |
| LDL-C: HDL-C (n=57) | 0.10 (-0.16, 0.35) | 0.45 | 1.04 | 0.15 (-0.15, 0.45) | 0.35 | 2.41 | 0.16 (-0.15, 0.47) | 0.33 | 2.96 |
| TG (mmol/L) (n=57) | -0.07 (-0.19, 0.05) | 0.26 | 2.27 | -0.08 (-0.22, 0.06) | 0.29 | 5.12 | -0.09 (-0.24, 0.06) | 0.24 | 7.05 |
| DBP (mmHg) (n=478) | 0.77 (-0.19, 1.72) | 0.12 | 0.52 | -0.37 (-1.44, 0.70) | 0.50 | 5.73 | -0.32 (-1.40, 0.75) | 0.55 | 6.12 |
| SBP (mmHg) (n=478) | 2.93 (1.70, 4.16) | <0.001 | 4.41 | 1.69 (0.31, 3.06) | 0.016 | 9.54 | 1.71 (0.33, 3.09) | 0.016 | 9.59 |
| UA (μmol/L) (n=53) | -9.74 (-37.65, 18.16) | 0.50 | 0.91 | -6.22 (-39.23, 26.79) | 0.71 | 1.25 | -7.85 (-41.75, 26.04) | 0.65 | 5.33 |

^a^ The *β* coefficient represents the estimated change in the CMRF outcome associated with a one-unit increase in the respective skill-related physical fitness indicator. Model 1 was an univariable linear regression model. Model 2 was a multivariable linear regression model and adjusted for potential confounders, including age, sex and BMI. Model 3 further adjusted for physical activity on the basis of model 2. Data of FPG and TG were analyzed after log transformation.

^b^ The number of participants in groups of vertical jump with other CMRFs was less than 20, and the sample size was too small for analysis.

Abbreviations: BMI, body mass index; CI, confidence interval; CMRF, cardiometabolic risk factors; DBP, diastolic blood pressure; FPG, fasting plasma glucose; HDL-C, high-density lipoprotein cholesterol; LDL-C, low-density lipoprotein cholesterol; non-HDL-C, non-high-density lipoprotein cholesterol; SBP, systolic blood pressure; TC, total cholesterol; TG, triglyceride; UA, uric acid.

**Supplemental Table 5.** Associations between health-related physical fitness indicators and CMRFs in subgroups by age in Chinese adults (N = 877). ^a^

| **Parameters** | **Model 1** | | | **Model 2** | | | **Model 3** | | |
| --- | --- | --- | --- | --- | --- | --- | --- | --- | --- |
|  | ***β* (95% CI)** | ***P*** | **R^2^ (%)** | ***β* (95% CI)** | ***P*** | **R^2^ (%)** | ***β* (95% CI)** | ***P*** | **R^2^ (%)** |
| **< 60 years** |  |  |  |  |  |  |  |  |  |
| **Body composition** |  |  |  |  |  |  |  |  |  |
| *Body fat mass (kg)* |  |  |  |  |  |  |  |  |  |
| FPG (mmol/L) (n=82) | -0.01 (-0.08, 0.05) | 0.66 | 0.25 | 0.01 (-0.16, 0.18) | 0.89 | 0.49 | -0.01 (-0.18, 0.17) | 0.94 | 4.56 |
| TC (mmol/L) (n=75) | -0.12 (-0.36, 0.11) | 0.32 | 1.37 | 0.18 (-0.47, 0.83) | 0.59 | 3.92 | 0.23 (-0.46, 0.93) | 0.51 | 7.79 |
| LDL-C (mmol/L) (n=73) | -0.08 (-0.30, 0.13) | 0.44 | 0.84 | 0.25 (-0.34, 0.83) | 0.41 | 4.23 | 0.27 (-0.36, 0.89) | 0.40 | 5.85 |
| non-HDL-C (mmol/L) (n=73) | -0.10 (-0.33, 0.13) | 0.38 | 1.11 | 0.11 (-0.53, 0.75) | 0.73 | 2.82 | 0.23 (-0.44, 0.90) | 0.50 | 8.40 |
| HDL-C (mmol/L) (n=73) | -0.02 (-0.09, 0.05) | 0.59 | 0.41 | 0.06 (-0.14, 0.26) | 0.56 | 1.88 | -0.001 (-0.213, 0.210) | 0.99 | 5.74 |
| TC: HDL-C (n=72) | -0.03 (-0.28, 0.21) | 0.80 | 0.09 | 0.12 (-0.57, 0.80) | 0.74 | 0.64 | 0.34 (-0.38, 1.07) | 0.35 | 5.80 |
| LDL-C: HDL-C (n=72) | -0.01 (-0.23, 0.20) | 0.90 | 0.02 | 0.27 (-0.33, 0.88) | 0.38 | 1.46 | 0.40 (-0.25, 1.05) | 0.23 | 3.41 |
| TG (mmol/L) (n=75) | 0.04 (-0.06, 0.13) | 0.46 | 0.75 | 0.05 (-0.22, 0.32) | 0.73 | 2.31 | 0.12 (-0.16, 0.41) | 0.40 | 7.12 |
| DBP (mmHg) (n=841) | 1.34 (0.65, 2.03) | <0.001 | 1.71 | 2.25 (0.83, 3.67) | 0.002 | 10.91 | 2.21 (0.79, 3.64) | 0.002 | 11.04 |
| SBP (mmHg) (n=841) | 2.53 (1.57, 3.49) | <0.001 | 3.09 | 2.54 (0.62, 4.46) | 0.010 | 16.82 | 2.64 (0.72, 4.56) | 0.007 | 17.26 |
| UA (μmol/L) (n=65) | -6.24 (-30.70, 18.22) | 0.62 | 0.40 | -15.17 (-78.07, 47.74) | 0.64 | 11.28 | -5.13 (-73.39, 63.13) | 0.88 | 14.85 |
| *Body fat percentage (%)* |  |  |  |  |  |  |  |  |  |
| FPG (mmol/L) (n=82) | -0.02 (-0.09, 0.04) | 0.53 | 0.50 | -0.10 (-0.28, 0.08) | 0.29 | 1.88 | -0.09 (-0.28, 0.10) | 0.36 | 5.59 |
| TC (mmol/L) (n=75) | -0.05 (-0.30, 0.21) | 0.72 | 0.18 | 0.08 (-0.60, 0.76) | 0.83 | 3.60 | 0.15 (-0.54, 0.83) | 0.68 | 7.44 |
| LDL-C (mmol/L) (n=73) | -0.03 (-0.26, 0.20) | 0.79 | 0.10 | 0.06 (-0.55, 0.68) | 0.84 | 3.33 | 0.10 (-0.52, 0.72) | 0.76 | 5.00 |
| non-HDL-C (mmol/L) (n=73) | -0.05 (-0.29, 0.20) | 0.71 | 0.19 | 0.01 (-0.66, 0.68) | 0.97 | 2.65 | 0.09 (-0.57, 0.76) | 0.78 | 7.89 |
| HDL-C (mmol/L) (n=73) | -0.001 (-0.078, 0.076) | 0.97 | 0 | 0.08 (-0.13, 0.28) | 0.47 | 2.14 | 0.06 (-0.15, 0.27) | 0.56 | 6.23 |
| TC: HDL-C (n=72) | -0.08 (-0.34, 0.19) | 0.58 | 0.45 | -0.03 (-0.75, 0.68) | 0.92 | 0.49 | 0.03 (-0.68, 0.75) | 0.93 | 4.57 |
| LDL-C: HDL-C (n=72) | -0.04 (-0.27, 0.20) | 0.75 | 0.14 | 0.05 (-0.59, 0.68) | 0.89 | 0.37 | 0.08 (-0.57, 0.72) | 0.82 | 1.35 |
| TG (mmol/L) (n=75) | 0.01 (-0.09, 0.12) | 0.83 | 0.07 | 0.11 (-0.17, 0.39) | 0.44 | 2.98 | 0.15 (-0.14, 0.43) | 0.31 | 7.53 |
| DBP (mmHg) (n=841) | -0.14 (-0.84, 0.56) | 0.70 | 0.02 | 2.84 (1.16, 4.52) | 0.001 | 11.05 | 2.81 (1.13, 4.49) | 0.001 | 11.19 |
| SBP (mmHg) (n=841) | -0.14 (-1.12, 0.85) | 0.78 | 0.01 | 2.67 (0.39, 4.95) | 0.022 | 16.68 | 2.73 (0.46, 5.01) | 0.019 | 17.09 |
| UA (μmol/L) (n=65) | -21.69 (-47.65, 4.28) | 0.11 | 4.08 | 7.19 (-60.33, 74.71) | 0.84 | 11.01 | 1.17 (-69.24, 71.57) | 0.97 | 14.83 |
| **Cardiorespiratory fitness** | |  |  |  |  |  |  |  |  |
| **Flexibility** |  |  |  |  |  |  |  |  |  |
| *Sit-and-reach (cm)* |  |  |  |  |  |  |  |  |  |
| FPG (mmol/L) (n=84) | -0.0005 (-0.0593, 0.0584) | 0.99 | 0 | -0.001 (-0.062, 0.059) | 0.97 | 0.28 | -0.002 (-0.063, 0.058) | 0.94 | 3.50 |
| TC (mmol/L) (n=77) | 0.14 (-0.09, 0.37) | 0.23 | 1.91 | 0.13 (-0.11, 0.37) | 0.29 | 3.77 | 0.12 (-0.11, 0.35) | 0.31 | 10.19 |
| LDL-C (mmol/L) (n=75) | 0.16 (-0.05, 0.36) | 0.14 | 2.93 | 0.15 (-0.06, 0.36) | 0.17 | 4.41 | 0.14 (-0.07, 0.35) | 0.18 | 7.87 |
| non-HDL-C (mmol/L) (n=75) | 0.13 (-0.10, 0.35) | 0.28 | 1.58 | 0.12 (-0.12, 0.35) | 0.33 | 3.01 | 0.10 (-0.12, 0.33) | 0.37 | 11.54 |
| HDL-C (mmol/L) (n=75) | 0.03 (-0.05, 0.10) | 0.49 | 0.66 | 0.02 (-0.05, 0.10) | 0.50 | 1.61 | 0.03 (-0.04, 0.10) | 0.45 | 5.03 |
| TC: HDL-C (n=74) | 0.07 (-0.18, 0.31) | 0.60 | 0.38 | 0.07 (-0.18, 0.33) | 0.58 | 0.49 | 0.06 (-0.19, 0.31) | 0.65 | 6.28 |
| LDL-C: HDL-C (n=74) | 0.07 (-0.15, 0.28) | 0.53 | 0.54 | 0.07 (-0.15, 0.29) | 0.54 | 0.58 | 0.06 (-0.16, 0.28) | 0.59 | 2.95 |
| TG (mmol/L) (n=77) | -0.03 (-0.13, 0.06) | 0.50 | 0.60 | -0.03 (-0.12, 0.07) | 0.59 | 2.43 | -0.03 (-0.13, 0.06) | 0.52 | 6.50 |
| DBP (mmHg) (n=862) | 0.11 (-0.58, 0.80) | 0.76 | 0.01 | 0.58 (-0.09, 1.24) | 0.09 | 10.01 | 0.57 (-0.10, 1.23) | 0.10 | 10.13 |
| SBP (mmHg) (n=862) | -0.17 (-1.13, 0.80) | 0.74 | 0.01 | 0.65 (-0.25, 1.54) | 0.16 | 16.58 | 0.72 (-0.18, 1.61) | 0.12 | 17.01 |
| UA (μmol/L) (n=67) | -4.81 (-28.49, 18.87) | 0.69 | 0.24 | -0.49 (-23.69, 22.72) | 0.97 | 9.16 | -0.31 (-23.46, 22.85) | 0.98 | 12.64 |
| **Muscular fitness** | |  |  |  |  |  |  |  |  |
| *Grip strength (kg)* |  |  |  |  |  |  |  |  |  |
| FPG (mmol/L) (n=86) | 0.02 (-0.04, 0.08) | 0.55 | 0.43 | 0.06 (-0.03, 0.16) | 0.20 | 2.36 | 0.06 (-0.04, 0.16) | 0.22 | 5.54 |
| TC (mmol/L) (n=79) | -0.07 (-0.31, 0.17) | 0.56 | 0.45 | 0.05 (-0.34, 0.44) | 0.81 | 2.56 | 0.05 (-0.34, 0.43) | 0.81 | 9.15 |
| LDL-C (mmol/L) (n=77) | -0.09 (-0.30, 0.13) | 0.43 | 0.82 | -0.02 (-0.36, 0.33) | 0.93 | 2.15 | -0.02 (-0.37, 0.33) | 0.91 | 5.75 |
| non-HDL-C (mmol/L) (n=77) | -0.03 (-0.26, 0.20) | 0.80 | 0.09 | 0.12 (-0.27, 0.50) | 0.55 | 2.33 | 0.13 (-0.25, 0.50) | 0.51 | 10.98 |
| HDL-C (mmol/L) (n=77) | -0.04 (-0.11, 0.04) | 0.33 | 1.27 | -0.06 (-0.18, 0.06) | 0.35 | 2.05 | -0.07 (-0.19, 0.05) | 0.27 | 5.22 |
| TC: HDL-C (n=76) | 0.08 (-0.17, 0,33) | 0.54 | 0.52 | 0.13 (-0.28, 0.54) | 0.52 | 0.72 | 0.17 (-0.24, 0.58) | 0.42 | 6.26 |
| LDL-C: HDL-C (n=76) | 0.02 (-0.20, 0.24) | 0.86 | 0.04 | 0.06 (-0.30, 0.42) | 0.74 | 0.23 | 0.08 (-0.29, 0.44) | 0.68 | 2.44 |
| TG (mmol/L) (n=79) | 0.06 (-0.04, 0.15) | 0.25 | 1.72 | 0.06 (-0.10, 0.21) | 0.49 | 2.64 | 0.07 (-0.09, 0.23) | 0.41 | 6.56 |
| DBP (mmHg) (n=877) | 2.18 (1.51, 2.86) | <0.001 | 4.38 | -0.19 (-1.31, 0.93) | 0.74 | 9.64 | -0.16 (-1.28, 0.96) | 0.78 | 9.76 |
| SBP (mmHg) (n=877) | 4.12 (3.19, 5.04) | <0.001 | 8.04 | 0.34 (-1.15, 1.84) | 0.65 | 16.75 | 0.29 (-1.20, 1.79) | 0.70 | 17.15 |
| UA (μmol/L) (n=69) | 17.35 (-6.52, 41.22) | 0.16 | 2.94 | -27.12 (-65.66, 11.42) | 0.17 | 13.47 | -22.89 (-62.37, 16.59) | 0.26 | 15.74 |
| **≥ 60 years** |  |  |  |  |  |  |  |  |  |
| **Body composition** |  |  |  |  |  |  |  |  |  |
| *Body fat mass (kg)* |  |  |  |  |  |  |  |  |  |
| FPG (mmol/L) (n=24) | 0.08 (-0.04, 0.21) | 0.20 | 7.36 | 0.07 (-0.31, 0.44) | 0.73 | 10.76 | -0.001 (-0.400, 0.398) | 1.00 | 17.96 |
| TC (mmol/L) (n=24) | 0.25 (-0.21, 0.72) | 0.30 | 4.92 | 1.46 (0.21, 2.72) | 0.034 | 21.50 | 1.45 (0.08, 2.82) | 0.05 | 22.42 |
| LDL-C (mmol/L) (n=24) | 0.35 (-0.04, 0.74) | 0.09 | 12.52 | 1.26 (0.18, 2.33) | 0.033 | 24.35 | 1.23 (0.07, 2.39) | 0.05 | 26.70 |
| non-HDL-C (mmol/L) (n=24) | 0.24 (-0.17, 0.64) | 0.26 | 5.68 | 1.17 (0.06, 2.27) | 0.05 | 18.85 | 1.14 (-0.06, 2.34) | 0.08 | 19.95 |
| HDL-C (mmol/L) (n=24) | 0.02 (-0.12, 0.16) | 0.81 | 0.28 | 0.30 (-0.09, 0.69) | 0.15 | 10.48 | 0.31 (-0.12, 0.74) | 0.17 | 10.87 |
| TC: HDL-C (n=24) | -0.01 (-0.45, 0.44) | 0.98 | 0 | 0.06 (-1.26, 1.38) | 0.93 | 0.10 | 0.04 (-1.41, 1.48) | 0.96 | 0.18 |
| LDL-C: HDL-C (n=24) | 0.24 (0.01, 0.46) | 0.05 | 16.09 | 0.50 (-0.16, 1.15) | 0.15 | 19.31 | 0.45 (-0.25, 1.16) | 0.22 | 21.66 |
| TG (mmol/L) (n=24) | 0.17 (0.05, 0.30) | 0.010 | 26.87 | 0.29 (-0.06, 0.64) | 0.13 | 28.68 | 0.30 (-0.09, 0.69) | 0.15 | 28.87 |
| DBP (mmHg) (n=46) | 1.54 (-1.33, 4.41) | 0.30 | 2.46 | 3.98 (-4.03, 11.99) | 0.34 | 6.89 | 4.07 (-3.90, 12.03) | 0.32 | 13.66 |
| SBP (mmHg) (n=46) | 1.59 (-3.44, 6.61) | 0.54 | 0.86 | 5.19 (-9.06, 19.45) | 0.48 | 2.57 | 6.09 (-8.45, 20.63) | 0.42 | 4.89 |
| UA (μmol/L) (n=23) | -12.16 (-64.64, 40.31) | 0.65 | 0.97 | -12.54 (-161.35, 136.27) | 0.87 | 6.21 | -5.26 (-167.01, 156.48) | 0.95 | 7.41 |
| *Body fat percentage (%)* |  |  |  |  |  |  |  |  |  |
| FPG (mmol/L) (n=24) | 0.06 (-0.05, 0.17) | 0.31 | 4.68 | 0.06 (-0.29, 0.42) | 0.73 | 10.76 | 0.03 (-0.35, 0.40) | 0.89 | 18.05 |
| TC (mmol/L) (n=24) | 0.11 (-0.29, 0.52) | 0.59 | 1.34 | 0.34 (-0.99, 1.67) | 0.62 | 2.29 | 0.20 (-1.26, 1.67) | 0.79 | 4.21 |
| LDL-C (mmol/L) (n=24) | 0.21 (-0.13, 0.56) | 0.24 | 6.15 | 0.46 (-0.68, 1.59) | 0.44 | 7.29 | 0.32 (-0.92, 1.55) | 0.62 | 10.28 |
| non-HDL-C (mmol/L) (n=24) | 0.12 (-0.23, 0.47) | 0.50 | 2.08 | 0.30 (-0.86, 1.45) | 0.62 | 2.79 | 0.17 (-1.10, 1.43) | 0.80 | 5.02 |
| HDL-C (mmol/L) (n=24) | -0.01 (-0.13, 0.11) | 0.88 | 0.10 | 0.04 (-0.35, 0.43) | 0.83 | 0.79 | 0.03 (-0.40, 0.47) | 0.88 | 0.90 |
| TC: HDL-C (n=24) | -0.01 (-0.40, 0.37) | 0.94 | 0.03 | 0.01 (-1.25, 1.27) | 0.99 | 0.07 | -0.02 (-1.42, 1.37) | 0.98 | 0.17 |
| LDL-C: HDL-C (n=24) | 0.17 (-0.03, 0.37) | 0.10 | 11.56 | 0.34 (-0.30, 0.98) | 0.31 | 14.90 | 0.26 (-0.44, 0.96) | 0.48 | 17.22 |
| TG (mmol/L) (n=24) | 0.14 (0.03, 0.24) | 0.020 | 22.27 | 0.17 (-0.18, 0.52) | 0.35 | 23.00 | 0.18 (-0.21, 0.56) | 0.38 | 23.10 |
| DBP (mmHg) (n=46) | 0.83 (-1.62, 3.28) | 0.51 | 0.99 | 4.58 (-2.92, 12.09) | 0.24 | 7.93 | 4.35 (-3.30, 12.01) | 0.27 | 14.17 |
| SBP (mmHg) (n=46) | 0.30 (-3.97, 4.58) | 0.89 | 0.04 | 0.14 (-13.37, 13.64) | 0.98 | 1.38 | 1.59 (-12.53, 15.70) | 0.83 | 3.41 |
| UA (μmol/L) (n=23) | -16.31 (-60.73, 28.11) | 0.48 | 2.41 | -11.70 (-152.73, 129.34) | 0.87 | 6.20 | -7.71 (-163.06, 147.64) | 0.92 | 7.44 |
| **Cardiorespiratory fitness** | |  |  |  |  |  |  |  |  |
| **Flexibility** |  |  |  |  |  |  |  |  |  |
| *Sit-and-reach (cm)* |  |  |  |  |  |  |  |  |  |
| FPG (mmol/L) (n=22) | -0.08 (-0.22, 0.06) | 0.28 | 5.89 | -0.10 (-0.27, 0.07) | 0.26 | 17.50 | -0.09 (-0.26, 0.08) | 0.33 | 23.10 |
| TC (mmol/L) (n=22) | 0.31 (-0.21, 0.82) | 0.26 | 6.41 | 0.29 (-0.34, 0.92) | 0.38 | 6.72 | 0.34 (-0.33, 1.02) | 0.33 | 8.00 |
| LDL-C (mmol/L) (n=22) | 0.24 (-0.20, 0.69) | 0.30 | 5.40 | 0.21 (-0.33, 0.74) | 0.46 | 9.97 | 0.25 (-0.33, 0.82) | 0.41 | 10.91 |
| non-HDL-C (mmol/L) (n=22) | 0.29 (-0.15, 0.73) | 0.21 | 7.84 | 0.28 (-0.26, 0.83) | 0.32 | 8.49 | 0.34 (-0.24, 0.92) | 0.27 | 10.29 |
| HDL-C (mmol/L) (n=22) | 0.01 (-0.14, 0.17) | 0.88 | 0.12 | 0.01 (-0.18, 0.20) | 0.92 | 0.44 | 0.01 (-0.20, 0.21) | 0.94 | 0.45 |
| TC: HDL-C (n=22) | 0.15 (-0.35, 0.64) | 0.56 | 1.69 | 0.22 (-0.39, 0.83) | 0.48 | 2.84 | 0.27 (-0.39, 0.92) | 0.44 | 3.79 |
| LDL-C: HDL-C (n=22) | 0.11 (-0.16, 0.37) | 0.45 | 2.90 | 0.09 (-0.23, 0.40) | 0.60 | 13.43 | 0.12 (-0.22, 0.45) | 0.50 | 15.30 |
| TG (mmol/L) (n=22) | 0.13 (-0.02, 0.28) | 0.10 | 12.80 | 0.12 (-0.05, 0.28) | 0.18 | 28.88 | 0.13 (-0.05, 0.30) | 0.18 | 29.30 |
| DBP (mmHg) (n=44) | 0.15 (-2.80, 3.09) | 0.92 | 0.02 | 0.39 (-2.94, 3.71) | 0.82 | 3.69 | 0.54 (-2.89, 3.98) | 0.76 | 4.14 |
| SBP (mmHg) (n=44) | -2.11 (-7.36, 3.15) | 0.44 | 1.45 | -1.98 (-7.99, 4.04) | 0.52 | 2.29 | -1.30 (-7.45, 4.85) | 0.68 | 4.88 |
| UA (μmol/L) (n=21) | 57.96 (8.85, 107.08) | 0.032 | 21.97 | 99.59 (50.73, 148.45) | <0.001 | 50.63 | 99.22 (46.37,152.07) | 0.002 | 50.64 |
| **Muscular Fitness** | |  |  |  |  |  |  |  |  |
| *Grip strength (kg)* |  |  |  |  |  |  |  |  |  |
| FPG (mmol/L) (n=24) | -0.10 (-0.21, 0.01) | 0.08 | 13.35 | -0.17 (-0.30, -0.04) | 0.022 | 31.38 | -0.16 (-0.30, -0.02) | 0.043 | 35.14 |
| TC (mmol/L) (n=24) | 0.02 (-0.40, 0.44) | 0.92 | 0.04 | 0.15 (-0.42, 0.72) | 0.62 | 2.34 | 0.20 (-0.41, 0.81) | 0.53 | 5.95 |
| LDL-C (mmol/L) (n=24) | -0.02 (-0.39, 0.35) | 0.90 | 0.07 | 0.10 (-0.39, 0.59) | 0.70 | 5.12 | 0.14 (-0.38, 0.66) | 0.60 | 10.44 |
| non-HDL-C (mmol/L) (n=24) | 0.04 (-0.32, 0.41) | 0.82 | 0.23 | 0.19 (-0.30, 0.68) | 0.46 | 4.25 | 0.24 (-0.28, 0.76) | 0.38 | 8.72 |
| HDL-C (mmol/L) (n=24) | -0.02 (-0.14, 0.10) | 0.74 | 0.52 | -0.04 (-0.21, 0.13) | 0.65 | 1.59 | -0.04 (-0.22, 0.14) | 0.68 | 1.73 |
| TC: HDL-C (n=24) | 0.12 (-0.27, 0.51) | 0.55 | 1.65 | 0.18 (-0.35, 0.71) | 0.52 | 2.20 | 0.20 (-0.38, 0.78) | 0.50 | 2.69 |
| LDL-C: HDL-C (n=24) | -0.01 (-0.22, 0.21) | 0.96 | 0.01 | 0.08 (-0.20, 0.36) | 0.58 | 11.76 | 0.11 (-0.18, 0.41) | 0.47 | 17.33 |
| TG (mmol/L) (n=24) | -0.04 (-0.17, 0.08) | 0.51 | 1.99 | 0.03 (-0.12, 0.18) | 0.68 | 20.23 | 0.04 (-0.13, 0.20) | 0.65 | 20.63 |
| DBP (mmHg) (n=45) | 1.94 (-0.78, 4.65) | 0.17 | 4.35 | 2.93 (-0.56, 6.41) | 0.11 | 10.94 | 3.25 (-0.33, 6.83) | 0.08 | 18.79 |
| SBP (mmHg) (n=45) | 0.24 (-4.68, 5.17) | 0.92 | 0.02 | 0.27 (-6.23, 6.76) | 0.94 | 1.41 | 1.19 (-5.73, 8.12) | 0.74 | 3.27 |
| UA (μmol/L) (n=23) | 26.44 (-17.82, 70.71) | 0.25 | 6.13 | 17.61 (-41.83, 77.05) | 0.57 | 7.71 | 15.16 (-49.43, 79.75) | 0.65 | 8.52 |

^a^ The *β* coefficient represents the estimated change in the CMRF outcome associated with a one-unit increase in the respective health-related physical fitness indicator. Model 1 was an univariable linear regression model. Model 2 was a multivariable linear regression model and adjusted for potential confounders, including age, sex and BMI. Model 3 further adjusted for physical activity on the basis of model 2. Data of FPG and TG were analyzed after log transformation.

Abbreviations: BMI, body mass index; CI, confidence interval; CMRF, cardiometabolic risk factors; DBP, diastolic blood pressure; FPG, fasting plasma glucose; HDL-C, high-density lipoprotein cholesterol; LDL-C, low-density lipoprotein cholesterol; non-HDL-C, non-high-density lipoprotein cholesterol; SBP, systolic blood pressure; TC, total cholesterol; TG, triglyceride; UA, uric acid.

**Supplemental Table 6.** Associations between skill-related physical fitness indicators and CMRFs in subgroups by age in Chinese adults (N = 875). ^a^

| **Parameters** | **Model 1** | | | **Model 2** | | | **Model 3** | | |
| --- | --- | --- | --- | --- | --- | --- | --- | --- | --- |
|  | ***β* (95% CI)** | ***P*** | **R^2^ (%)** | ***β* (95% CI)** | ***P*** | **R^2^ (%)** | ***β* (95% CI)** | ***P*** | **R^2^ (%)** |
| **< 60 years** |  |  |  |  |  |  |  |  |  |
| **Balance** |  |  |  |  |  |  |  |  |  |
| *One-leg standing time (s)* | |  |  |  |  |  |  |  |  |
| FPG (mmol/L) (n=83) | -0.01 (-0.08, 0.07) | 0.86 | 0.04 | -0.01 (-0.09, 0.06) | 0.77 | 0.37 | -0.04 (-0.12, 0.04) | 0.33 | 5.43 |
| TC (mmol/L) (n=76) | 0.28 (-0.03, 0.59) | 0.08 | 4.18 | 0.25 (-0.07, 0.57) | 0.13 | 5.50 | 0.24 (-0.10, 0.57) | 0.17 | 11.52 |
| LDL-C (mmol/L) (n=74) | 0.19 (-0.09, 0.47) | 0.19 | 2.40 | 0.16 (-0.13, 0.45) | 0.29 | 3.76 | 0.14 (-0.17, 0.45) | 0.38 | 6.91 |
| non-HDL-C (mmol/L) (n=74) | 0.24 (-0.07, 0.54) | 0.13 | 3.16 | 0.21 (-0.11, 0.53) | 0.20 | 4.14 | 0.21 (-0.12, 0.54) | 0.22 | 12.52 |
| HDL-C (mmol/L) (n=74) | 0.05 (-0.05, 0.14) | 0.33 | 1.32 | 0.04 (-0.06, 0.14) | 0.41 | 1.71 | 0.03 (-0.08, 0.13) | 0.64 | 3.97 |
| TC: HDL-C (n=73) | 0.01 (-0.31, 0.34) | 0.94 | 0.01 | 0.01 (-0.33, 0.35) | 0.96 | 0.17 | 0.05 (-0.31, 0.41) | 0.79 | 5.72 |
| LDL-C: HDL-C (n=73) | 0.02 (-0.27, 0.30) | 0.90 | 0.02 | 0.01 (-0.29, 0.30) | 0.96 | 0.13 | 0.03 (-0.29, 0.34) | 0.88 | 2.41 |
| TG (mmol/L) (n=76) | -0.05 (-0.18, 0.08) | 0.44 | 0.81 | -0.03 (-0.17, 0.10) | 0.61 | 2.93 | -0.03 (-0.17, 0.11) | 0.65 | 6.63 |
| DBP (mmHg) (n=861) | -1.68 (-2.36, -1.00) | <0.001 | 2.69 | -1.14 (-1.80, -0.48) | <0.001 | 10.73 | -1.18 (-1.84, -0.52) | <0.001 | 10.99 |
| SBP (mmHg) (n=861) | -2.44 (-3.38, -1.50) | <0.001 | 2.92 | -1.41 (-2.30, -0.53) | 0.002 | 17.45 | -1.35 (-2.24, -0.47) | 0.003 | 17.72 |
| UA (μmol/L) (n=66) | -23.47 (-53.54, 6.60) | 0.13 | 3.53 | -21.77 (-51.29, 7.75) | 0.15 | 16.13 | -17.97 (-50.16, 14.22) | 0.28 | 17.04 |
| **Reaction time** |  |  |  |  |  |  |  |  |  |
| *Choice reaction time (s)* | |  |  |  |  |  |  |  |  |
| FPG (mmol/L) (n=86) | 0.01 (-0.04, 0.05) | 0.82 | 0.06 | 0.01 (-0.04, 0.06) | 0.76 | 0.45 | 0.01 (-0.04, 0.06) | 0.65 | 3.99 |
| TC (mmol/L) (n=79) | 0.17 (-0.01, 0.35) | 0.07 | 4.09 | 0.20 (0.01, 0.39) | 0.043 | 7.69 | 0.18 (-0.02, 0.37) | 0.08 | 12.95 |
| LDL-C (mmol/L) (n=77) | 0.20 (0.04, 0.36) | 0.014 | 7.75 | 0.23 (0.06, 0.40) | 0.008 | 11.16 | 0.23 (0.06, 0.40) | 0.012 | 13.89 |
| non-HDL-C (mmol/L) (n=77) | 0.15 (-0.02, 0.33) | 0.09 | 3.71 | 0.18 (-0.01, 0.37) | 0.06 | 6.41 | 0.14 (-0.05, 0.33) | 0.14 | 13.12 |
| HDL-C (mmol/L) (n=77) | 0.01 (-0.05, 0.07) | 0.74 | 0.15 | 0.01 (-0.05, 0.07) | 0.67 | 1.11 | 0.03 (-0.04, 0.09) | 0.40 | 4.50 |
| TC: HDL-C (n=76) | 0.10 (-0.09, 0.29) | 0.31 | 1.42 | 0.12 (-0.08, 0.33) | 0.24 | 2.03 | 0.08 (-0.13, 0.29) | 0.48 | 6.07 |
| LDL-C: HDL-C (n=76) | 0.14 (-0.03, 0.30) | 0.11 | 3.46 | 0.16 (-0.02, 0.33) | 0.08 | 4.18 | 0.14 (-0.04, 0.32) | 0.14 | 5.21 |
| TG (mmol/L) (n=79) | -0.02 (-0.09, 0.06) | 0.66 | 0.25 | -0.02 (-0.10, 0.06) | 0.58 | 2.40 | -0.04 (-0.12, 0.04) | 0.32 | 6.96 |
| DBP (mmHg) (n=875) | 0.26 (-0.49, 1.00) | 0.50 | 0.05 | 0.45 (-0.28, 1.18) | 0.23 | 9.74 | 0.53 (-0.21, 1.27) | 0.16 | 9.94 |
| SBP (mmHg) (n=875) | 1.38 (0.35, 2.42) | 0.009 | 0.78 | 1.71 (0.74, 2.68) | <0.001 | 17.82 | 1.63 (0.65, 2.61) | 0.001 | 18.07 |
| UA (μmol/L) (n=69) | -1.04 (-19.67, 17.58) | 0.91 | 0.02 | 5.84 (-13.09, 24.78) | 0.55 | 11.43 | 5.11 (-14.44, 24.67) | 0.61 | 14.37 |
| **≥ 60 years** |  |  |  |  |  |  |  |  |  |
| **Balance** |  |  |  |  |  |  |  |  |  |
| *One-leg standing time (s)* | |  |  |  |  |  |  |  |  |
| FPG (mmol/L) (n=23) | -0.63 (-1.57, 0.30) | 0.20 | 7.77 | -0.75 (-1.71, 0.21) | 0.14 | 20.12 | -1.13 (-2.60, 0.33) | 0.15 | 28.40 |
| TC (mmol/L) (n=22) | 1.27 (-2.12, 4.65) | 0.47 | 2.61 | 1.54 (-2.19, 5.27) | 0.43 | 4.32 | 2.46 (-3.71, 8.62) | 0.45 | 5.38 |
| LDL-C (mmol/L) (n=22) | 1.41 (-1.61, 4.44) | 0.37 | 4.04 | 1.75 (-1.47, 4.97) | 0.30 | 11.66 | 2.14 (-3.20, 7.47) | 0.44 | 12.26 |
| non-HDL-C (mmol/L) (n=22) | 1.31 (-1.64, 4.27) | 0.39 | 3.65 | 1.62 (-1.61, 4.86) | 0.34 | 6.58 | 2.75 (-2.57, 8.06) | 0.33 | 8.62 |
| HDL-C (mmol/L) (n=22) | -0.05 (-1.07, 0.97) | 0.93 | 0.04 | -0.08 (-1.21, 1.05) | 0.89 | 0.47 | -0.29 (-2.16, 1.58) | 0.77 | 1.05 |
| TC: HDL-C (n=22) | 1.43 (-1.89, 4.75) | 0.41 | 3.46 | 1.48 (-2.20, 5.16) | 0.44 | 3.50 | 3.73 (-2.22, 9.68) | 0.24 | 8.90 |
| LDL-C: HDL-C (n=22) | 0.73 (-1.11, 2.57) | 0.44 | 2.96 | 0.93 (-0.96, 2.82) | 0.35 | 16.47 | 1.20 (-1.92, 4.31) | 0.46 | 18.25 |
| TG (mmol/L) (n=22) | 0.09 (-0.97, 1.15) | 0.87 | 0.14 | 0.28 (-0.77, 1.32) | 0.61 | 21.77 | 0.66 (-1.05, 2.38) | 0.46 | 23.41 |
| DBP (mmHg) (n=43) | -4.28 (-8.68, 0.12) | 0.06 | 8.13 | -4.31 (-8.80, 0.17) | 0.07 | 12.50 | -4.85 (-9.26, -0.44) | 0.038 | 21.28 |
| SBP (mmHg) (n=43) | -4.41 (-12.29, 3.46) | 0.28 | 2.86 | -4.39 (-12.58, 3.80) | 0.30 | 3.59 | -4.13 (-12.50, 4.23) | 0.34 | 6.44 |
| UA (μmol/L) (n=21) | 418.42 (111.96, 724.87) | 0.015 | 27.37 | 415.86 (77.19, 754.53) | 0.028 | 27.59 | 827.80 (314.98, 1340.62) | 0.006 | 45.11 |
| **Reaction time** |  |  |  |  |  |  |  |  |  |
| *Choice reaction time (s)* | |  |  |  |  |  |  |  |  |
| FPG (mmol/L) (n=23) | 0.14 (0.06, 0.22) | 0.003 | 35.88 | 0.14 (0.06, 0.22) | 0.004 | 42.87 | 0.14 (0.06, 0.22) | 0.004 | 50.80 |
| TC (mmol/L) (n=23) | 0.26 (-0.09, 0.60) | 0.16 | 9.32 | 0.24 (-0.12, 0.61) | 0.21 | 9.92 | 0.24 (-0.15, 0.63) | 0.25 | 10.97 |
| LDL-C (mmol/L) (n=23) | 0.23 (-0.06, 0.53) | 0.14 | 10.13 | 0.20 (-0.11, 0.52) | 0.22 | 13.77 | 0.19 (-0.14, 0.52) | 0.27 | 16.38 |
| non-HDL-C (mmol/L) (n=23) | 0.21 (-0.09, 0.51) | 0.18 | 8.39 | 0.20 (-0.12, 0.51) | 0.24 | 9.39 | 0.19 (-0.15, 0.53) | 0.29 | 10.62 |
| HDL-C (mmol/L) (n=23) | 0.05 (-0.06, 0.15) | 0.40 | 3.40 | 0.05 (-0.06, 0.16) | 0.40 | 4.11 | 0.05 (-0.07, 0.17) | 0.42 | 4.38 |
| TC: HDL-C (n=23) | 0.03 (-0.31, 0.37) | 0.87 | 0.13 | 0.03 (-0.33, 0.40) | 0.86 | 0.25 | 0.03 (-0.36, 0.42) | 0.88 | 0.38 |
| LDL-C: HDL-C (n=23) | 0.12 (-0.06, 0.30) | 0.22 | 7.09 | 0.09 (-0.09, 0.27) | 0.35 | 16.16 | 0.08 (-0.11, 0.27) | 0.42 | 18.47 |
| TG (mmol/L) (n=23) | 0.01 (-0.09, 0.12) | 0.81 | 0.27 | -0.01 (-0.11, 0.09) | 0.81 | 21.36 | -0.01 (-0.12, 0.10) | 0.83 | 21.40 |
| DBP (mmHg) (n=45) | 0.40 (-1.44, 2.25) | 0.67 | 0.43 | 0.35 (-1.49, 2.20) | 0.71 | 5.31 | 0.30 (-1.58, 2.18) | 0.76 | 11.81 |
| SBP (mmHg) (n=45) | 4.19 (1.26, 7.11) | 0.008 | 15.46 | 4.19 (1.21, 7.17) | 0.009 | 16.62 | 3.96 (0.82, 7.09) | 0.018 | 17.43 |
| UA (μmol/L) (n=22) | -24.40 (-61.03, 12.23) | 0.21 | 7.85 | -23.81 (-62.09, 14.47) | 0.24 | 12.95 | -22.14 (-62.30, 18.02) | 0.30 | 16.83 |

^a^ The *β* coefficient represents the estimated change in the CMRF outcome associated with a one-unit increase in the respective skill-related physical fitness indicator. Model 1 was an univariable linear regression model. Model 2 was a multivariable linear regression model and adjusted for potential confounders, including age, sex and BMI. Model 3 further adjusted for physical activity on the basis of model 2. Data of FPG and TG were analyzed after log transformation.

Abbreviations: BMI, body mass index; CI, confidence interval; CMRF, cardiometabolic risk factors; DBP, diastolic blood pressure; FPG, fasting plasma glucose; HDL-C, high-density lipoprotein cholesterol; LDL-C, low-density lipoprotein cholesterol; non-HDL-C, non-high-density lipoprotein cholesterol; SBP, systolic blood pressure; TC, total cholesterol; TG, triglyceride; UA, uric acid.


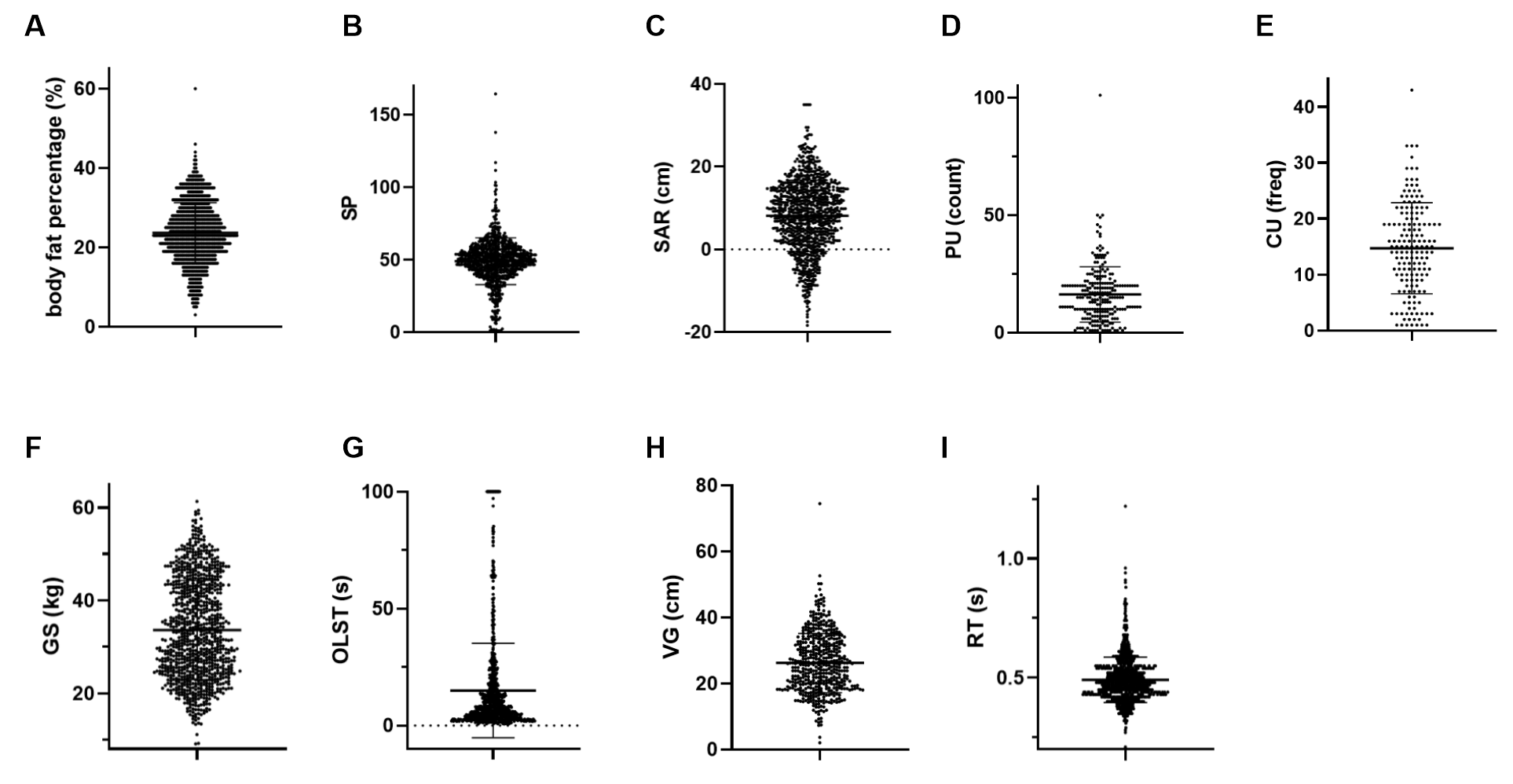


**Supplemental Figure 1.** Scatter plot for the distribution of physical fitness indicators in a community-based cross-sectional study of Chinese adults. (A) body fat percentage; (B) SP; (C) SAR; (D) PU; (E) CU; (F) GS; (G) OLST; (H) VG; (I) RT. Abbreviations: CU, curl-up; GS, grip strength; OLST, one-leg standing time; PU, push-up; RT, choice reaction time; SAR, sit-and-reach; SP, step index; VG, vertical jump.
